# Quantile-dependent expressivity of postprandial lipemia

**DOI:** 10.1371/journal.pone.0229495

**Published:** 2020-02-26

**Authors:** Paul T. Williams

**Affiliations:** Lawrence Berkeley National Laboratory, Berkeley, CA, United States of America; The Ohio State University, UNITED STATES

## Abstract

**Purpose:**

“Quantile-dependent expressivity” describes an effect of the genotype that depends upon the level of the phenotype (e.g., whether a subject’s triglycerides are high or low relative to its population distribution). Prior analyses suggest that the effect of a genetic risk score (GRS) on fasting plasma triglyceride levels increases with the percentile of the triglyceride distribution. Postprandial lipemia is well suited for testing quantile-dependent expressivity because it exposes each individual’s genotype to substantial increases in their plasma triglyceride concentrations. Ninety-seven published papers were identified that plotted mean triglyceride response vs. time and genotype, which were converted into quantitative data. Separately, for each published graph, standard least-squares regression analysis was used to compare the genotype differences at time *t* (dependent variable) to average triglyceride concentrations at time *t* (independent variable) to assess whether the genetic effect size increased in association with higher triglyceride concentrations and whether the phenomenon could explain purported genetic interactions with sex, diet, disease, BMI, and drugs.

**Results:**

Consistent with the phenomenon, genetic effect sizes increased (P≤0.05) with increasing triglyceride concentrations for polymorphisms associated with *ABCA1*, *ANGPTL4*, *APOA1*, *APOA2*, *APOA4*, *APOA5*, *APOB*, *APOC3*, *APOE*, *CETP*, *FABP2*, *FATP6*, *GALNT2*, *GCKR*, *HL*, *IL1b*, *LEPR*, *LOX-1*, *LPL*, *MC4R*, *MTTP*, *NPY*, *SORT1*, *SULF2*, *TNFA*, *TCF7L2*, and *TM6SF2*. The effect size for these polymorphisms showed a progressively increasing dose-response, with intermediate effect sizes at intermediate triglyceride concentrations. Quantile-dependent expressivity provided an alternative interpretation to their interactions with sex, drugs, disease, diet, and age, which have been traditionally ascribed to gene-environment interactions and genetic predictors of drug efficacy (i.e., personalized medicine).

**Conclusion:**

Quantile-dependent expressivity applies to the majority of genetic variants affecting postprandial triglycerides, which may arise because the impaired functionalities of these variants increase at higher triglyceride concentrations. Purported gene-drug interactions may be the manifestations of quantile-dependent expressivity, rather than genetic predictors of drug efficacy.

## Introduction

The majority of a person’s day is spent in the postprandial state, which is characterized by the elevation of triglyceride-rich lipoproteins (TRL) [1]. Zilversmit initially proposed that postprandial lipemia contributes significantly to coronary heart disease [2].

Postprandial lipemia is the consequence of the relative rates of intestinal fat absorption, TRL synthesis and lipolysis, intervascular lipid transfers, and plasma clearance of TRL remnants [1]. Following a fatty meal, long-chain fatty acids are absorbed by the intestines and esterified to form triglycerides that are then incorporated into chylomicrons for release into the circulation. The triglycerides are subsequently removed from circulation by lipoprotein lipase (LPL) which is a rate-limiting hydrolytic enzyme located on the vascular endothelium. This requires apolipoprotein (apo) CII, a cofactor for LPL that is carried on the chylomicrons after being received from high-density lipoproteins (HDL). The chylomicrons are called remnant particles when approximately 90% of their original triglyceride content has been hydrolyzed. During this process there is a loss of apo CIII (an inhibitor of TRL catabolism and clearance) and gain of apo E (a ligand for the receptor-mediated hepatic uptake of the remnants). Hepatic lipase hydrolyzes some of the remaining triglycerides, which helps facilitate hepatic receptor uptake of the remnant particles by exposing their apo E. LPL bound to the chylomicron remnants also assists with their receptor uptake.

Quantile-dependent expressivity describes an effect of the genotype on the phenotype that depends upon the level of the phenotype [3]. Using quantile regression, the relationship of plasma triglyceride levels to its genetic risk score (GRS) has been shown to increase with the percentile of the triglyceride distribution, i.e., the effect of the GRS depends upon whether an individual has high or low triglycerides relative to the others in the population [3]. Postprandial lipemia is particularly well suited for testing quantile-dependent expressivity because it represents the exposure of each individual’s genotype to substantial increases of their plasma triglyceride concentrations. Specifically, quantile-dependent expressivity hypothesizes that the triglyceride difference between genotypes (dependent variable in a simple linear regression analyses) will increase with the average triglyceride concentrations (independent variable) over the time course of the postprandial response. This means there will be a larger genetic effect size at hypertriglyceridemic (i.e., postprandial state) than at normotriglyceridemic concentrations (fasting state).

To test this hypothesis, quantitative data were extracted from the postprandial response graphs from 97 published papers out of 128 identified as potentially relevant through Pubmed search of genetics and postprandial triglycerides or oral fat tolerance test and literature cited within each paper (S1 Table) [4–131]. Included among these were several articles that were identified in preparation for another paper on gene-environment interactions of fasting triglyceride and their cited references. Studies were only considered if they presented graphs of the postprandial response by genotypes, provided information from which total and genotype-specific triglyceride levels could be calculated for at least four time point, and whose subjects were not selected for their pathological lipemic response. For each published response graph, plots were created for the genetic effect at each time point “t” vs. the average triglyceride concentrations at time t. Their analyses show that the majority of genetic variants affecting the postprandial triglyceride response have effect sizes that change depending upon the average triglyceride concentration at the time of measurement. Quantile-dependent expressivity provides an alternative explanation for: 1) purported genetic interactions of postprandial triglycerides with sex, diet, and disease, and 2) purported genetic markers of fenofibrate efficacy (i.e., personalized medicine).

## Results

**Fig 1:**
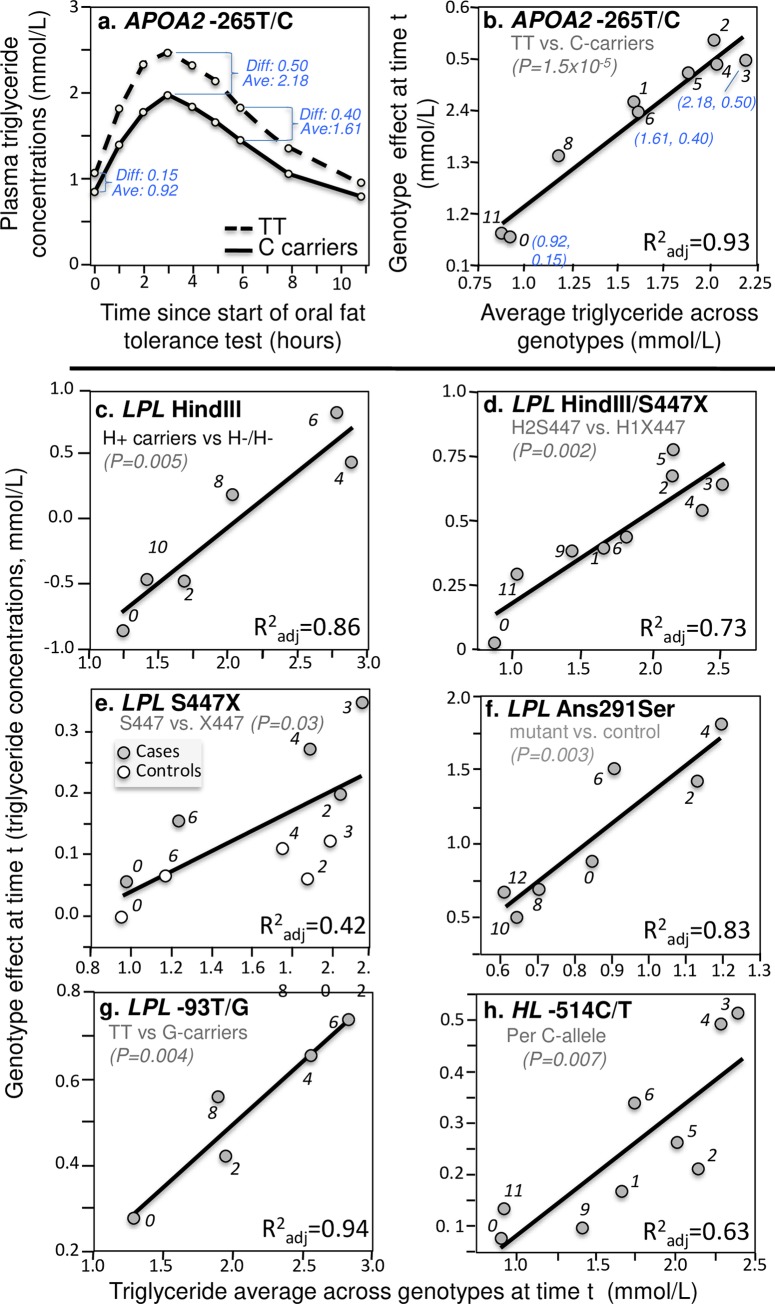
Quantile-dependent expressivity plots for postprandial triglyceride responses by *APOA2*, *HL*, and *LPL* polymorphisms. Panels (a) and (b) illustrate the methodology: (a) the re-rendering of the published triglyceride response to an oral fat tolerance test by *APOA2* -265T/C genotypes (rs5082) [25], from which is produced: (b) its *quantile-dependent expressivity plot* showing the linear relationship between the genotype differences (dependent variable) vs. the average triglyceride values (independent variable) at each time point “t” and its significance level. The lower panels present quantile-dependent expressivity plots derived from figures by: (c) Reiber et al. for 27 H+/+ and H+/- vs. 5 H-/- patients for the *LPL* intron 8 HindIII polymorphism (rs320) [103]; (d) López-Miranda et al. for 26 H2S447 vs. 15 H1X447 haplotypes (rs328) [68]; (e) Humphries et al. for 70.4% H+S447 and 19.2% H-S447 vs. 10.4% H-X447 male haplotypes (rs328) [49]; (f) Pimstone et al. for three Asn291Ser mutations of the *LPL* gene vs. five controls (rs268) [99]; (g) Talmud et al. for 70 TT homozygotes vs. 25 G-allele carriers of the -93T/G polymorphism in the *LPL* promoter region (rs1800590) [117]; and (h) Gómez et al. for 26 CC, 22 CT, and 3 TT of the -514C/T polymorphism in the promoter region of the hepatic lipase (*HL*) gene (rs1800588) [40]. The numerical labels refer to time (“0” is fasting).

The primary analyses are graphical as illustrated in Fig 1A and 1B. Fig 1A (upper left panel) is a re-rendering of Delgado-Lista et al.’s graph [25] of the triglyceride response following an oral fat tolerance test by *APOA2* -265T/C genotypes (rs5082). For each genotype, average triglyceride concentrations are presented for the fasting state at time 0, and the postprandial states at 1, 2, …, 6, 8.5 and 11 hours thereafter. The average triglyceride concentration across genotypes, and average triglyceride difference between genotypes, were determined for each time point (e.g., 0.92 and 0.15 mmol/L at time zero, respectively, 2.18 and 0.50 mmol/L at 3 hours, and 1.61 and 0.40 mmol/L at 6 hours) and used to create the quantile-dependent expressivity graph of Fig 1B. Specifically, Fig 1B plots the triglyceride differences between genotypes (the Y or dependent variable) vs. the average triglyceride value (the X or independent variable) at each time t to assess the genetic effect size as a function of triglyceride concentrations. The nine points (identified by time) exhibit a strong linear relationship as demonstrated by their proximity to their least-squares regression line, corresponding adjusted R-square of 0.93, and the statistical significance of the slope (P = 1.5x10^-5^). Therefore, consistent with the hypothesis of its quantile-dependent expressivity, the *APOA2* -265T/C effect size increased with increasing plasma triglyceride concentrations.

**Fig 2:**
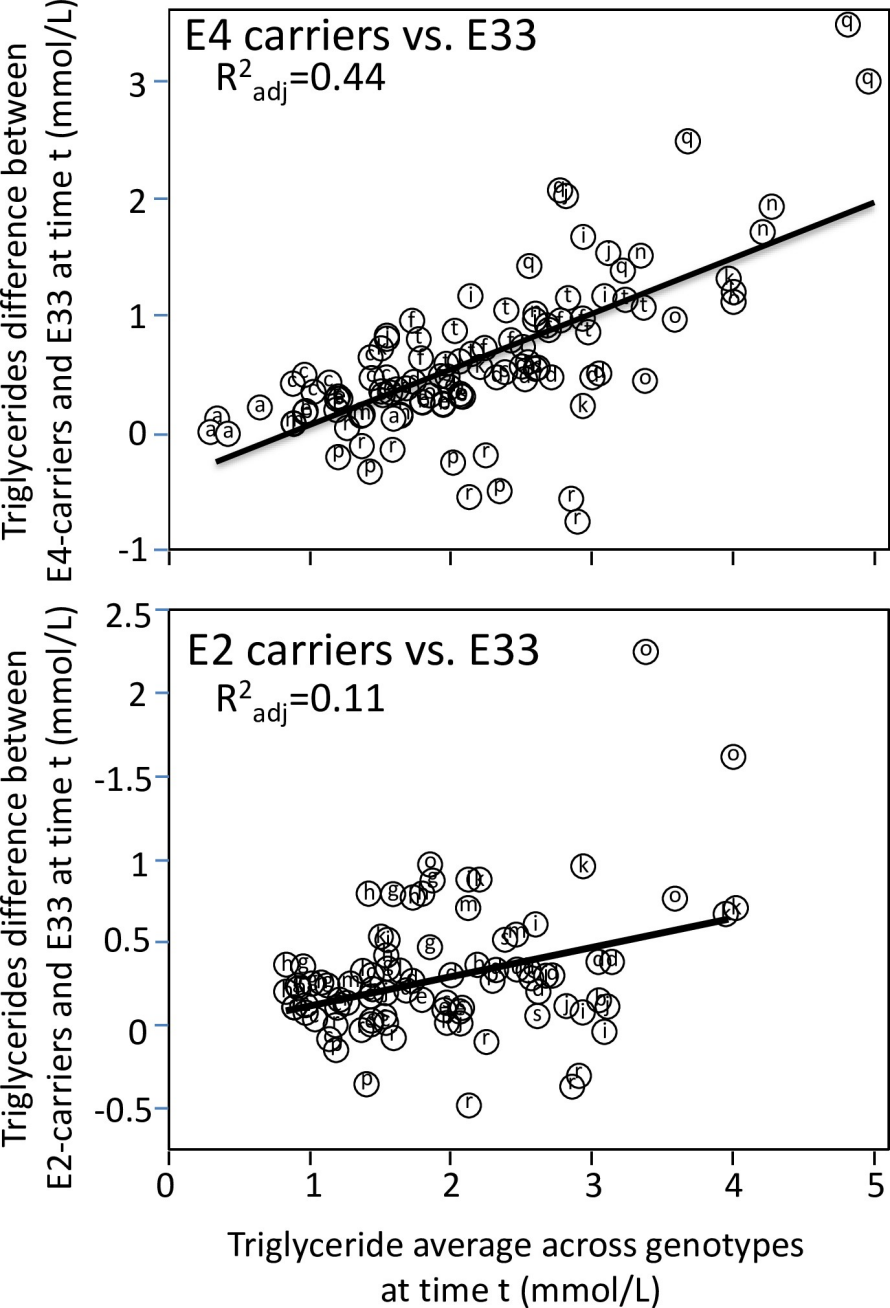
Quantile-dependent expressivity plots for postprandial triglyceride responses by *APOE* genotypes. Quantile-dependent expressivity showing increasing genetic effect of apo E4- and E2-carriers vs. E33 homozygotes with increasing average triglyceride levels. Data estimated from the published excursion plots from 10,876 measurements in E33, 4682 measurements in E4-carriers, and 2311 measurements in E2-carriers. Point source coded as follows: a) Bergeron et al. [7], b) Boerwinkle et al. [9], c) Brown et al. [11], d) Carvalho-Wells et al. [18], e) Dallongeville et al. [22], f) Dart et al. [23], g) Erkkila et al. at 8 weeks [30], h) Erkkilä et al. at baseline [30], i) Ferreira et al. for intensive training [32], j) Ferreira et al. for moderate training [32], k) Ferreira et al. for sedentary activity [32], l) Irvin et al. post-treatment [51], m) Irvin et al. pre-treatment [51], n) Kobayashi et al. [60], o) Nikkilä et al. cases [85], p) Nikkilä et al. controls [85], q) Reiber et al. [103], r) Reznik et al. [104], s) Vansant et al. [122], and t) Wolever et al. [129].

Apo E isoforms are the most-reported genetic modifier of postprandial triglyceride concentrations, with heightened responses reported for both E2-carriers [22, 51,104] and E4-carriers [7,11,18,22,23,60,104]. Apo E is thought to be a cofactor of VLDL catabolism, and reverse cholesterol transport, and is located on the surface of remnant particles where it is recognized by remnant receptors [18]. Fig 2 shows the differences between E4-carriers and E33 homozygotes (dependent variable) increased an average of 0.48 mmol/L for each one mmol/L increment in average triglyceride levels (0.39 mmol/L slope when weighted by study sample sizes) and differences between E2-carriers and E33 homozygotes increased an average of 0.12 mmol/L for each one mmol/L increment in average triglyceride levels (the same as when weighted by study sample size).

The LPL enzyme plays a central role in TRL catabolism by hydrolyzing triglyceride, and it participates in hepatic TRL clearance via the LDL receptor-related protein. The 447X variant of the Serine447-Stop S447X (rs328) polymorphism has a 2 amino acid truncation on LPL’s carboxyl-terminal domain, which enhances binding of cell surface receptors to TRL. The S*447X* polymorphism is in complete linkage disequilibrium with the *HindIII* polymorphism (rs320) [49,68]. Fig 1C–1G display the quantile dependence expressivity of these and other lipoprotein lipase genetic variants on postprandial triglyceride concentrations. Fig 1G shows that quantile-dependent expressivity also affected the postprandial triglyceride levels associated with the -93G allele of *LPL* promoter (rs1800590) [117]. Quantile-dependent expressivity is evident for the -514C/T polymorphism in the promoter region of the hepatic lipase (*HL*) gene (rs1800588, Fig 1H) [40].

**Fig 3:**
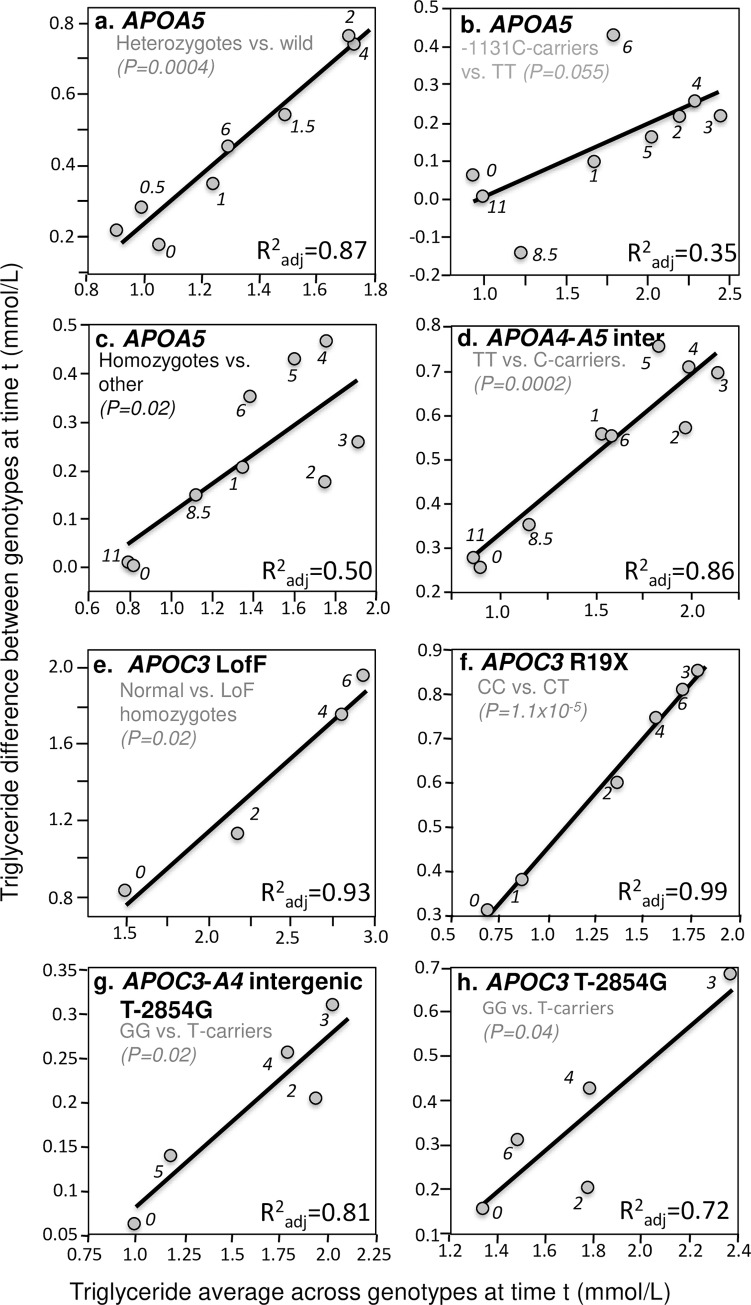
Quantile-dependent expressivity plots for postprandial triglyceride responses by *APOA4*, *APOA5*, and *APOC3* polymorphisms. Derived from the postprandial response figures published by: (a) Zemánková et al. for ten heterozygotes (seven -1131T->C and three 56C>G heterozygotes, rs662799 and rs3135506, respectively) vs. 20 wild type carriers of the *APOA5* gene [131]; (b) Moreno et al. for 12 C-allele carriers vs. 39 TT patients for the -1131T>C polymorphism of the *APOA5* promoter region (rs662799) [79]; (c) Moreno-Luna et al. for 65 patients with the haplotype defined by homozygous for the major alleles of -1131T>C (rs662799), c.-3A>G (rs651821), c56C>G (rs3135506), IVS3+476G>A (rs2072560) and c.1259T>C (rs2266788) vs. 21 others [80]; (d) Delgado-Lista et al. for 30 TT, 42 TC and 16 CC genotypes from the intergenic region between *APOA4* and *APOA5* (rs1263177) [27]; (e) Saleheen et al. for seven normal vs. six *APOC3* loss of function homozygotes (rs76353203) [108]; (f) Pollin et al. for 763 CC vs. 39 CT for the R19X mutation of the *APOC3* gene (rs76353203) [100]; (g) Waterworth et al. for 284 TT, 348 TG, and 85GG patients for the T-2854G polymorphism (rs1263177) within the *APOC3*-*APOA4* intergenic region [126]; and (h) Woo et al. for 18 GG vs. 42 T-carriers for this polymorphism within the *APOC3*-*APOA4* intergenic region [130].

The *APOA5* gene is the strongest genetic determinant of plasma triglyceride concentrations [132]. It is thought to participate in hepatic synthesis and secretion of TRL, stimulate LPL activity, and facilitate receptor-mediated clearance of TRL [133]. Located in the promoter region of *APOA5* gene, -1131T>C (rs662799) might lower apo AV levels by down-regulating *APOA5* mRNA translation [134]. Jang et al. [56], Moreno et al. [79], Martin et al. [71], Cardona et al. [16], and Zemánková et al. [131] all report significantly greater postprandial triglyceride increases in carriers of the C allele than in TT homozygote. Fig 3A–3C illustrate quantile-dependent expressivity for *APOA5* genetic variants. In addition, Cardona et al. reported that C-carriers had triglyceride concentrations that were 55% higher at baseline, 61% higher after 3 hours postprandial, and 68% higher after 4 hours postprandial than in TT homozygotes [16] (their results are examined in the Discussion Section in the context of drug treatment). Fig 3D shows the rs1263177 polymorphism in the intergenic region between *APOA4* and *APOA5*, which is thought to be a nonfunctional variant, also exhibits quantile-dependent expressivity [27].

Apo C-III is a component of TRLs that inhibits apoE-mediated remnant clearance [19]. Multiple *APOC3* genetic variants exhibit quantile-dependent expressivity. These include the Saleheen et al. report of lower fasting and postprandial triglycerides in loss of function (LofF) *APOC3* p.Arg19Ter homozygotes (rs76353203, Fig 3E) [108], the Pollin et al. report of heterozygous carriers of a null mutation (R19X) in the *APOC3* gene that express half the apoC-III of non-carriers (rs76353203, Fig 3F) [100], and the Waterworth et al. [126] (Fig 3G) and Woo et al. [130] (Fig 3H) reports of the T-2854G site that lies in the *APOC3*-*APOA4* intergenic region within an *APOC3* and *APOA4* enhancer element.

**Fig 4:**
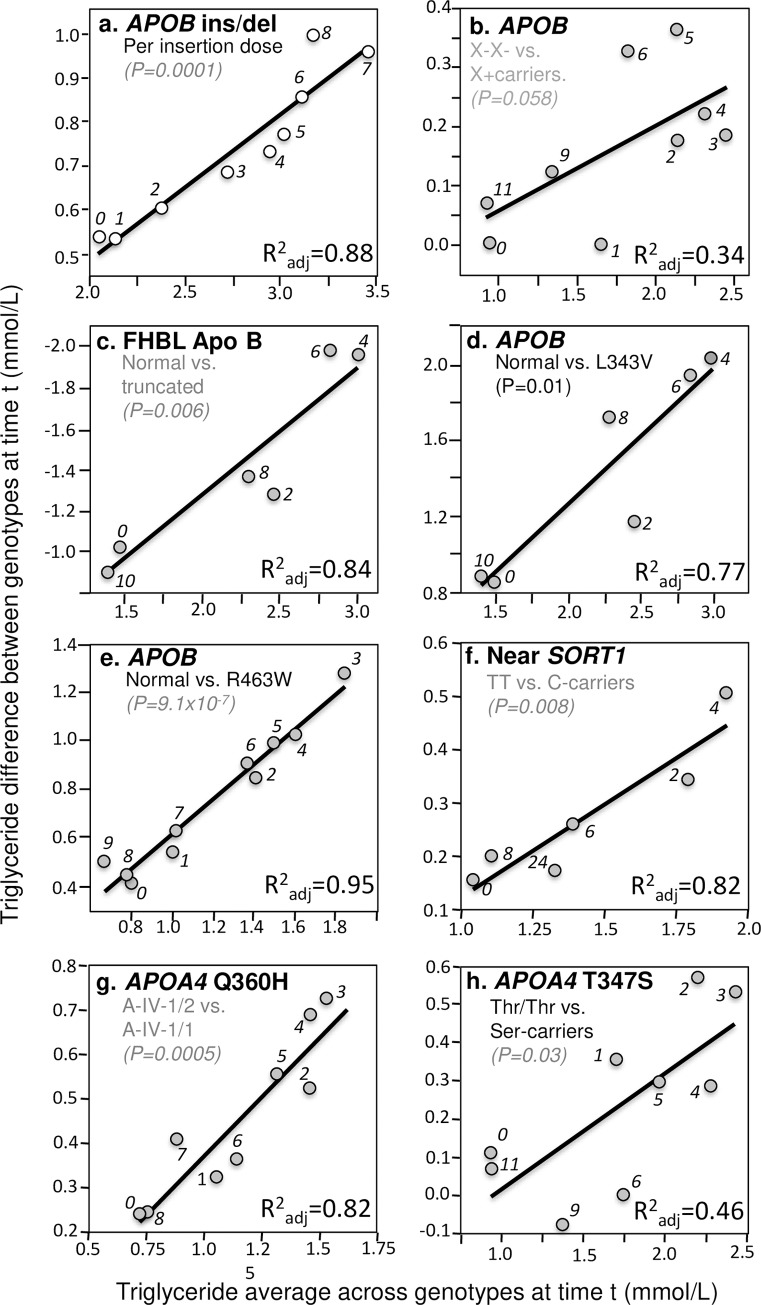
Quantile-dependent expressivity plots for postprandial triglyceride responses by *APOA4*, *APOB*, and *SORT1* polymorphisms. Derived from the postprandial response figures published by: (a) Vimaleswaran et al. for 52 del/del, 70 del/ins, and 25 ins/ins patients for the *APOB* insertion/deletion (ins/del) polymorphism (rs17240441) [124]; (b) Lopez-Miranda et al. for 31 carriers of the X+ allele vs. 20 X- homozygotes for the XbaI restriction site adjacent to *APOB* (rs693) [67]; (c) Hooper et al. for 10 normolipidemic controls vs. six heterozygous (three apoB-6.9, one apoB-25.8, and two apoB-40.3) familial hypobetalipoproteinemia (FHBL) patients [47]; (d) Hooper et al. for 10 healthy controls v. three heterogeneous *APOB* L343V mutations for FHBL [48]; (e) Noto et al. for six healthy controls vs. four heterogeneous *APOB* R463W mutations [86]; (f) Connors et al. for 15 TT homozygotes vs. 15 C-allele carriers for rs646776 variant of the 1p13 locus (near *SORT1*) [20]; (g) Hockey et al. for 14 A-IV-2 heterozygous vs. 14 A-IV-1 homozygous and for the *APOA4* Q360H polymorphism (rs5110) [45]; and (h) Ostos et al. for 36 Thr/Thr homozygote vs. 14 Ser-allele carriers for the *APOA4* 347Ser polymorphism [91].

**Fig 5:**
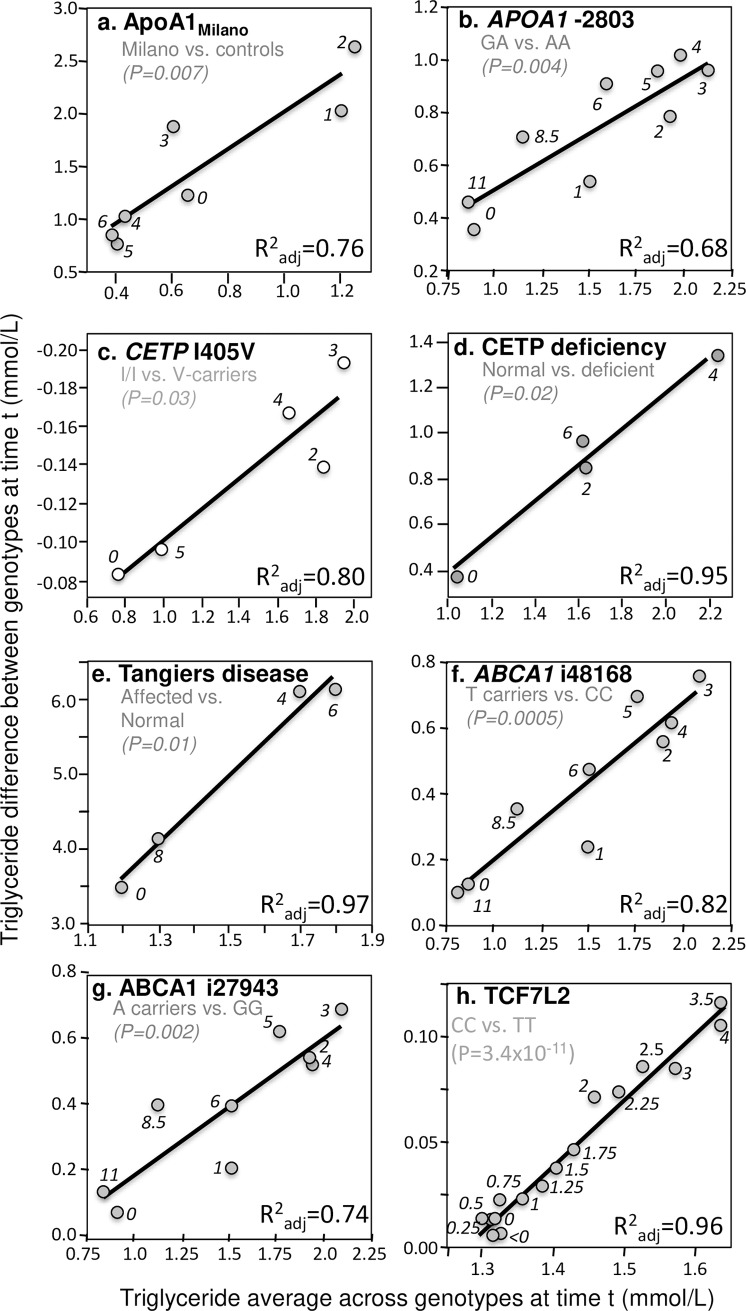
Quantile-dependent expressivity plots for postprandial triglyceride responses by *ABCA1*, *APOA1*, *CETP*, and *TCF7L2* polymorphisms. Derived from the postprandial response figures published by: (a) Calabresi et al. for 6 heterozygous apo A-I_Milano_ vs. 6 matched controls [13]; (b) Delgado-Lista et al. for 32 GA vs. 9 AA genotypes for the -2803G/A polymorphisn in the *APOA1* promoter region (rs2727784) [27]; (c) Gudnason et al. for 60 I/I, 55 I/V, and 27 V/V men for the I405V *CETP* polymorphism in men homozygous for the TaqIB B2 allele (rs5882) [42]; (d) Inazu et al. for 10 normal vs. 4 *CETP* deficient patients (mutations of intron 14(+1) G-to-A (14A) and D442G) [50]; (e) Kolovou et al. for five Tangier disease patients (3 homozygotes, 2 heterozygotes) vs. 25 normal male controls [61]; (f) Delgado-Lista et al. for 65 T-carriers vs. 23 CC homozygotes for the i48168 variant of the *ABCA1* gene (rs4149272) [26]; (g) Delgado-Lista et al. for 67 A-allele carriers vs. 15 GG homozygotes vs. for the i27943 variant (rs2575875) of the *ABCA1* gene [26]; and (h) Engelbrechtsen et al. for 31 CC vs. 31 TT homozygotes of the *TCF7L2* polymorphism (rs7903146) [29].

**Fig 6:**
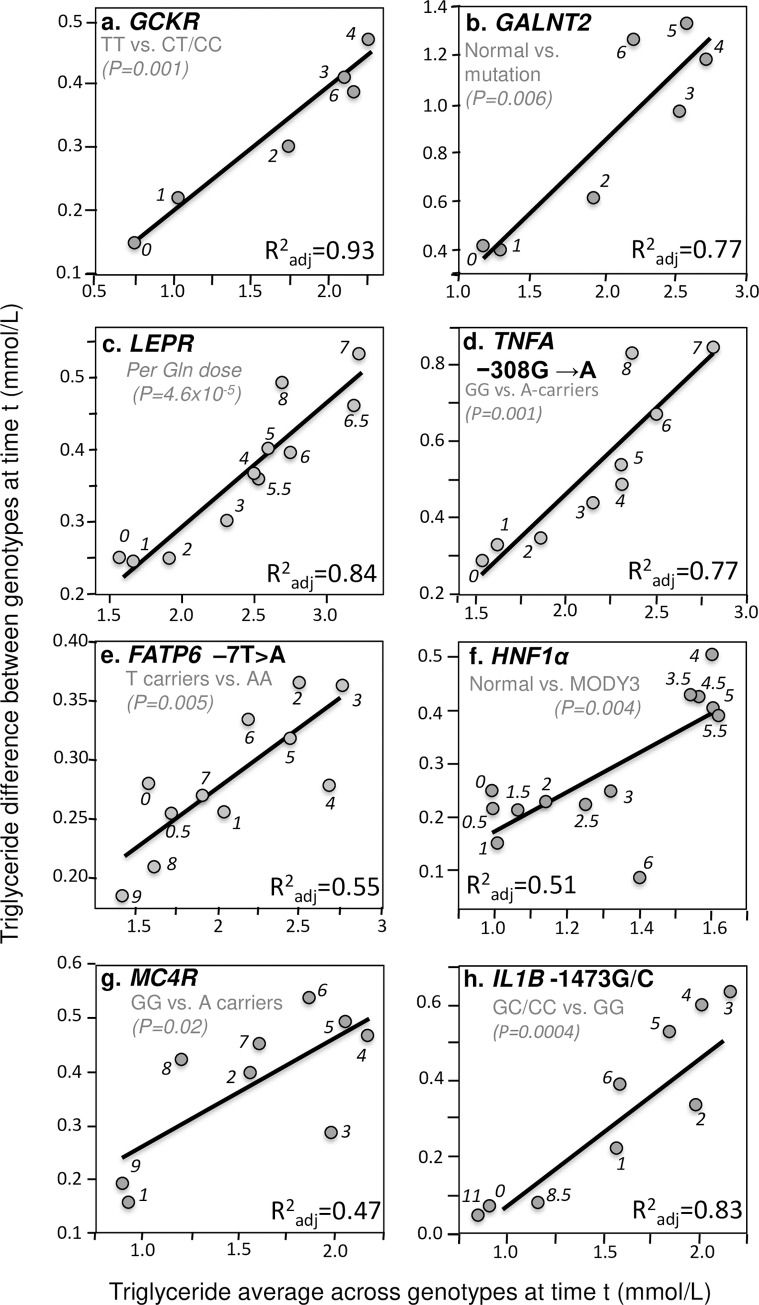
Quantile-dependent expressivity plots for postprandial triglycerides by *GALNT2*, *GCKR*, *lL1B*, *LEPR*, *MC4R* and *TNFA* polymorphisms. Derived from the postprandial response figures published by: (a) Shen et al. in 80 TT homozygotes vs. 690 carriers of the C allele of the P446L polymorphism in the *GCKR* gene (rs1260326) [112]; (b) Holleboom et al. for 4 normal and 4 patients with c.941A>C, p.D314A mutations in the *GALNT2* gene [46]; (c) Jackson et al. for 71 patients with zero, 122 with one, and 38 patients with two doses of the Gln allele for the Gln223Arg polymorphism (rs1137101) in the common leptin receptor (*LEPR*) gene [52]; (d) Jackson et al. for 64 carriers of the A allele vs. 162 GG homozygotes for the *TNFA* −308 G/A polymorphism (rs1800629) [54]; (e) Auinger et al. 583 T carriers vs. 102 AA homozygotes for the *FATP6* –7T>A polymorphism (rs2526246) [6]; (f) St-Jean et al. for 9 normal vs. 5 genotypically confirmed Mature Onset Diabetes of the Young type 3 (MODY3) patients (two C.872insC and three P.arg159trp patients) [115]; (g) Perez-Martinez et al. for 53 GG homozygotes vs. 35 A-carriers for rs12970134 polymorphism near the *MC4R* gene [97], and (h) Delgado-Lista et al. for 43 carriers of the C allele vs. 45 GG homozygotes of the -1473G/C polymorphism (rs1143623) in the *lL1B* promoter region [28].

**Fig 7:**
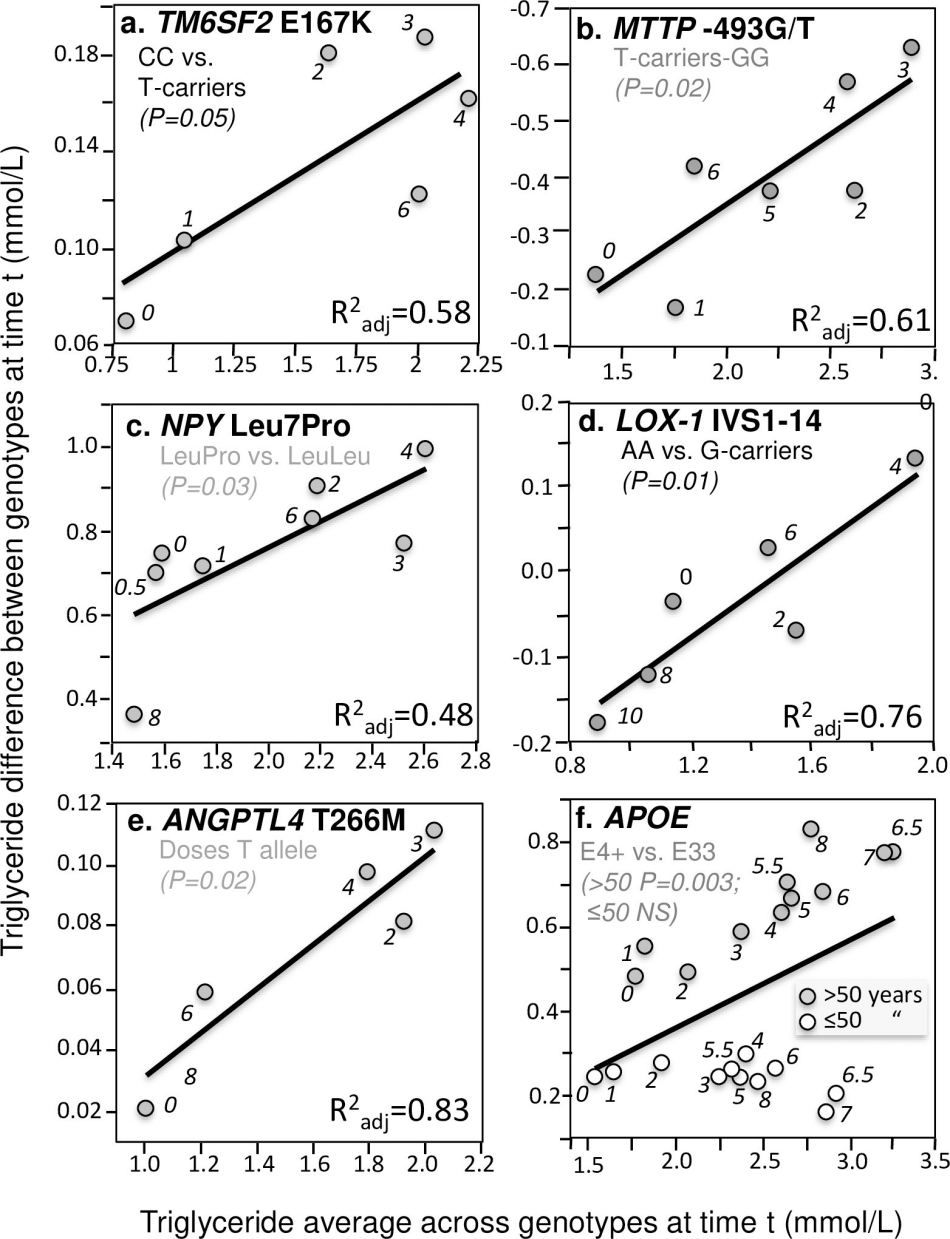
Quantile-dependent expressivity plots for postprandial triglycerides by *ANGPTL4*, *APOE*, *LOX-1*, *MTTP*, *NPY*, and *TM6SF2* polymorphisms. Derived from the postprandial response figures published by: (a) O’Hare et al. for 853 CC homozygotes vs.130 T-carriers for the *TM6SF2* loss-of-function variant (rs58542926) [87]; (b) Lundahl et al. for 24 GG homozygote vs. 36 carriers of the T-allele of the -493G/T polymorphism of the microsomal triglyceride transfer protein (*MTTP*, rs1800591) (P = 0.02) [69]; (c) Schwab et al. for 7 LeuPro heterozygotes vs. 7 LeuLeu homozygotes for the Leu7Pro polymorphism of the neuropeptide Y (*NPY*, rs16139) gene [109]; (d) Musso et al. for 26 AA homozygotes vs. 54 G-carriers of the lectin-like oxidized LDL receptor-1 (*LOX-1*) IVS4-14 A/G polymorphism in the pooled sample of NASH and healthy control patients [82]; (e) Talmud et al. for 1355 TT, 1108 TM, and 262 MM genotypes of *ANGPTL4* T266M (rs1044250) [118]; (f) Carvalho-Wells et al. for 143 E33 and 64 E4 carriers verifying their different postprandial response by age when matched for average triglyceride concentrations [18].

**Fig 8:**
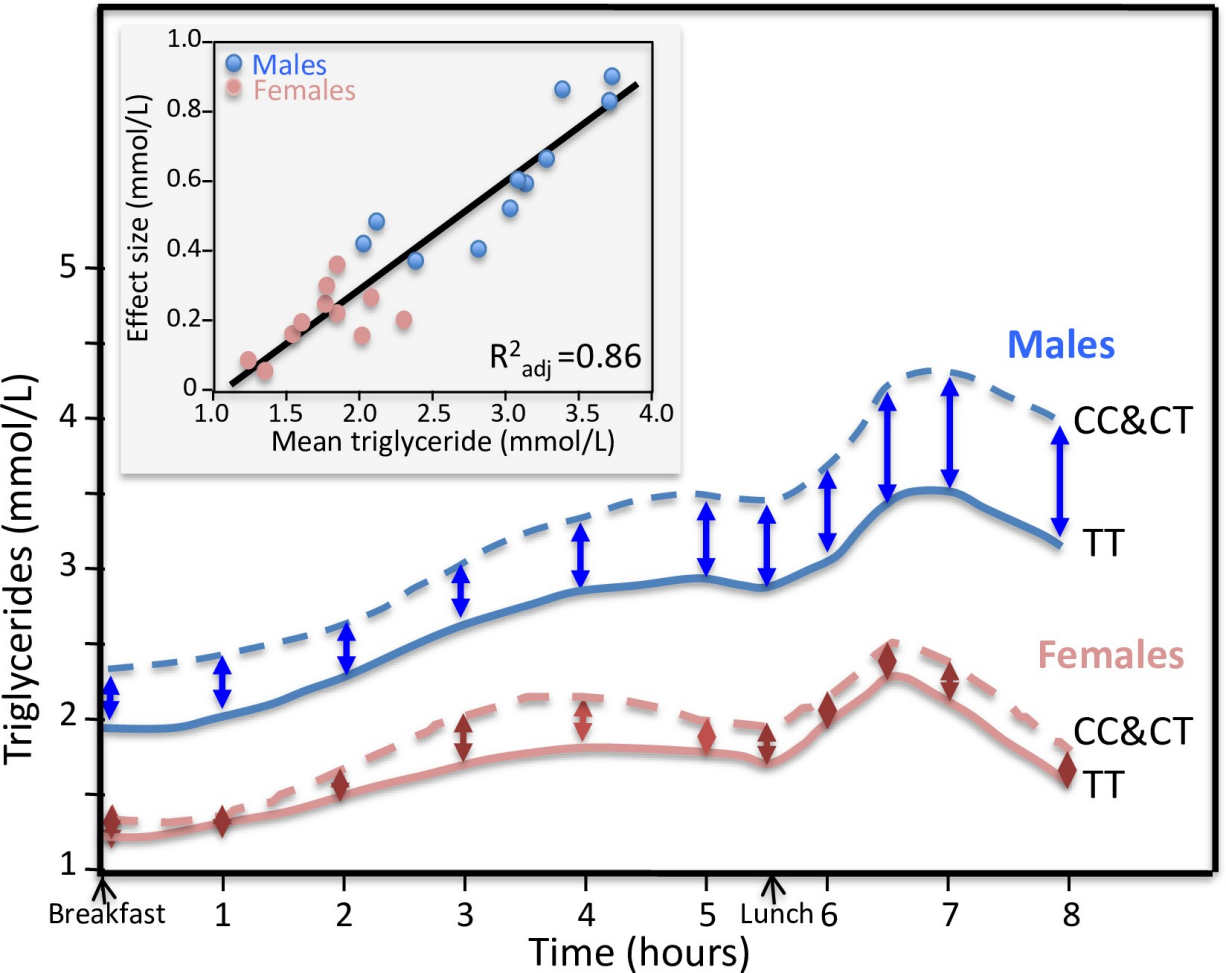
Sex-specific postprandial triglyceride responses in C-carrier vs. TT homozygotes of the *APOA5*–1131 T>C polymorphism. Re-rendering of the sex-specific postprandial triglyceride response published by Olano-Martin et al. [88]. The insert presents the quantile-dependent expressivity plot showing that males and females represent largely nonoverlapping triglyceride concentrations over which higher mean triglyceride concentrations predict increasing larger effect size between the C-carriers and TT homozygotes (P = 3.9x10^-10^).

**Fig 9:**
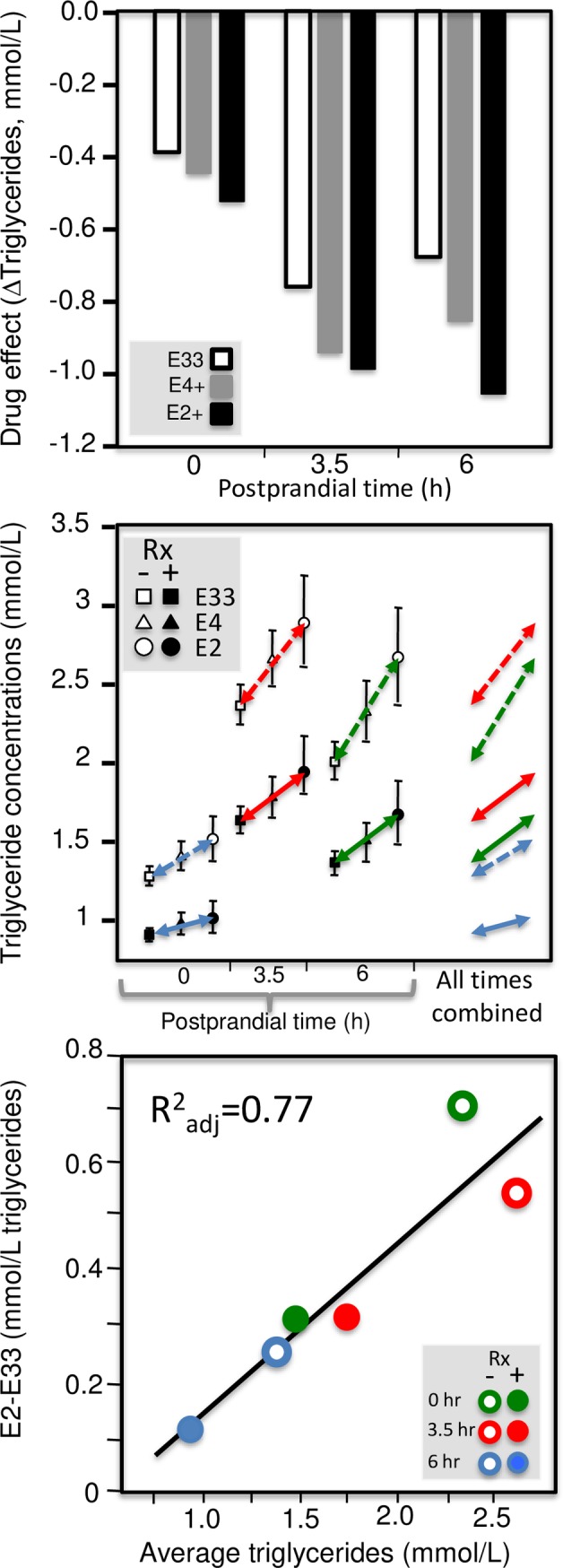
Quantile-dependent expressivity plots for pre- and post-fenofibrate treated postprandial triglyceride responses by *APOE* genotypes. Using data presented by Irvin et al. [51]: pre vs. post fenofibrate treated triglyceride concentrations by genotype (upper panel); genotype-specific mean triglyceride concentrations by treatment and genotype by time since meal (middle panel); quantile-dependent expressivity plot of the E2-E33 effect size vs. average triglyceride concentrations (bottom panel), suggesting the effect size is largely attributable to its relationship to mean triglyceride concentrations.

**Fig 10:**
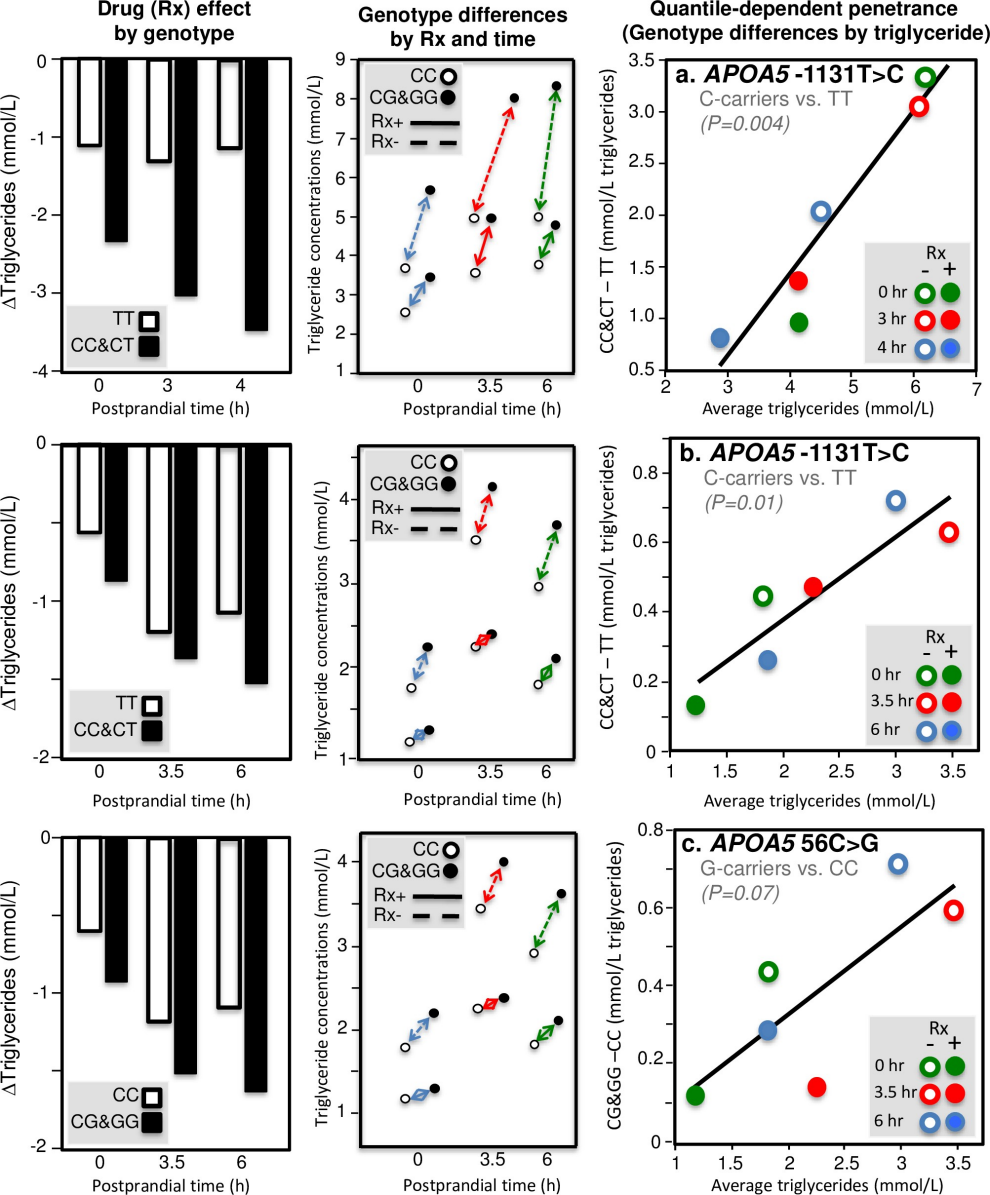
Quantile-dependent expressivity plots for pre- and post-fenofibrate treated postprandial triglyceride responses by *APOA5* genotypes. Derived from data presented by Cardona et al. for the -1131T>C of the *APOA5* gene (top row) [16] and Lai et al. for the -1131T>C (middle row) and 56C>G polymorphisms (bottom row) of the *APOA5* gene [63]. Left column presents pre vs. post fenofibrate treated triglyceride concentrations by genotype; center column present genotype-specific mean triglyceride concentrations by treatment and genotype by time since meal, and right column present quantile-dependent expressivity plot of the genetic effect size vs. average triglyceride concentrations, suggesting the effect sizes are largely attributable to their relationship to overall mean triglyceride concentrations.

**Fig 11:**
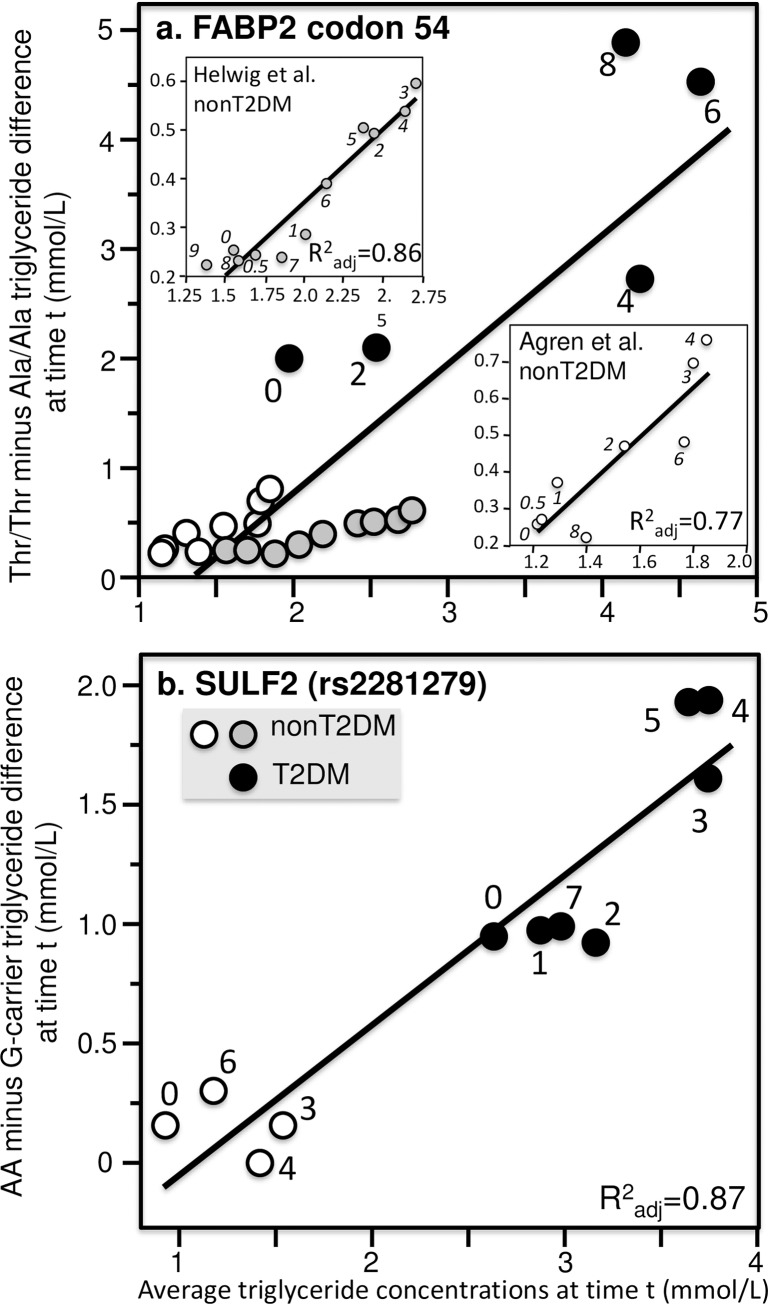
Quantile-dependent expressivity plots for postprandial triglyceride responses by *FABP2* and *SULF2* polymorphisms. (a) Derived from the postprandial response figures published by Helwig et al. for 360 AlaAla, 287 AlaThr, and 53 ThrThr nondiabetic patients [44] (shaded circles, P = 2.2x10^-5^), Agren et al. for 7 AlaAla and 8 ThrThr nondiabetic patients [4] (open circles, P = 0.003), and Georgopoulos et al. for 9 T2DM AlaAla and 6 T2DM ThrThr (P = 0.10) [37] of the codon 54 polymorphism of the *FABP2* gene (solid black circles rs1799883). Significance of the combined data: P = 8.1x10^-8^. (b) Matikainen et al. for 22 AA and 46 carriers of the G allele in nondiabetics (P = 0.54) [74] and Hassing et al. for 11 AA and 18 carriers of the G-allele in T2DM (P = 0.007) of the *SULF2* rs2281279 polymorphism [43] (combined data: P = 6.3x10^-6^).

**Fig 12:**
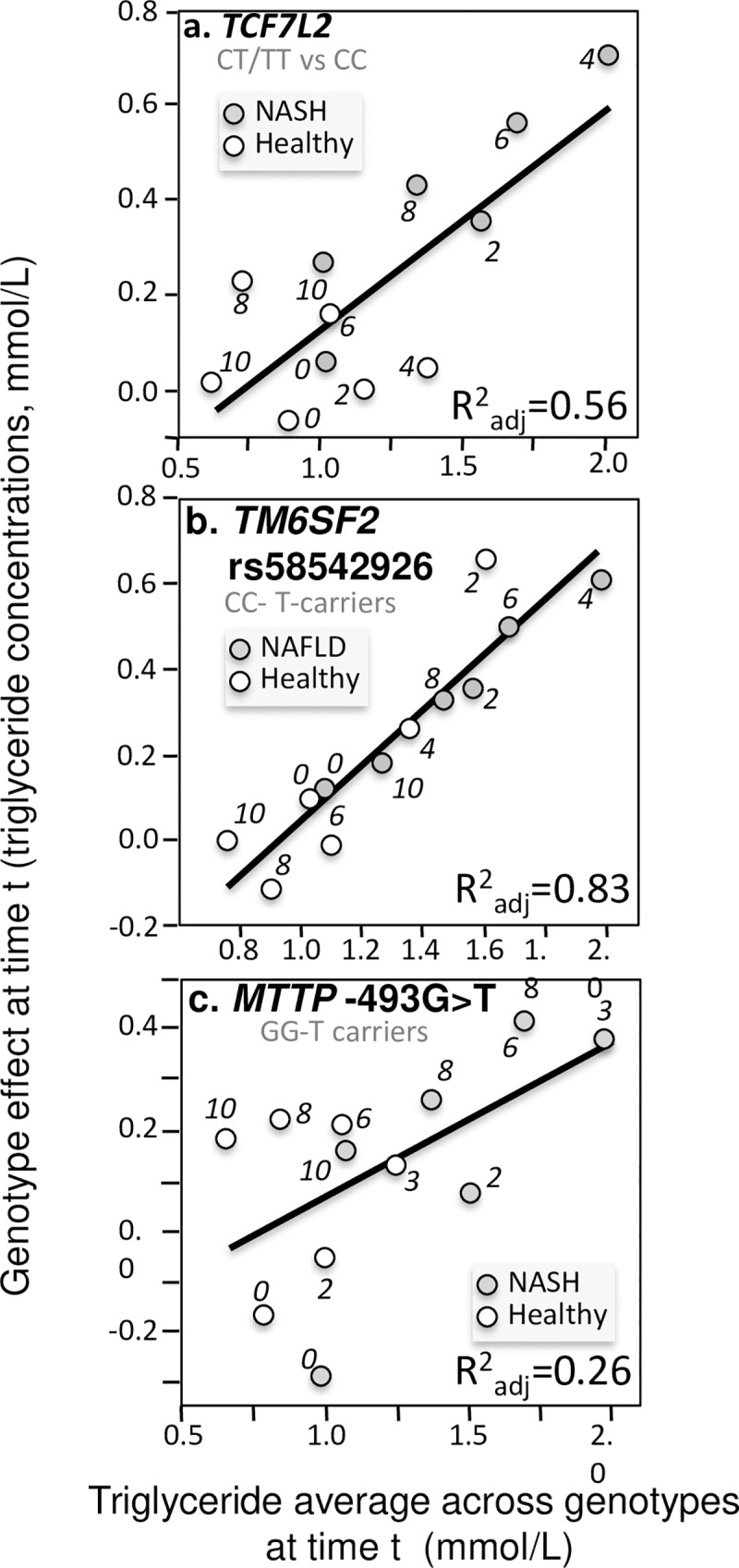
Quantile-dependent expressivity plots for postprandial triglyceride responses by *TCF7L2*, *TM6SF2*, and *MTTP* polymorphisms. Derived from the postprandial responses in NAFLD and non-NAFLD patients published by: a) Musso et al. for 38 T-carriers vs. 30 CC homozygotes of the rs7903146 polymorphism in the *TCF7L2* gene (P = 0.003) [81]; b) Musso et al. for 853 CC homozygotes vs. 130 T-carriers for the *TM6SF2* loss-of-function variant (rs58542926, P = 2.5x10^-5^) [83]; c) Gambino et al. for 32 GG homozygotes vs. 24 T-carriers for the -493 G/T polymorphism in the *MTTP* gene (P = 0.05) [35].

**Fig 13:**
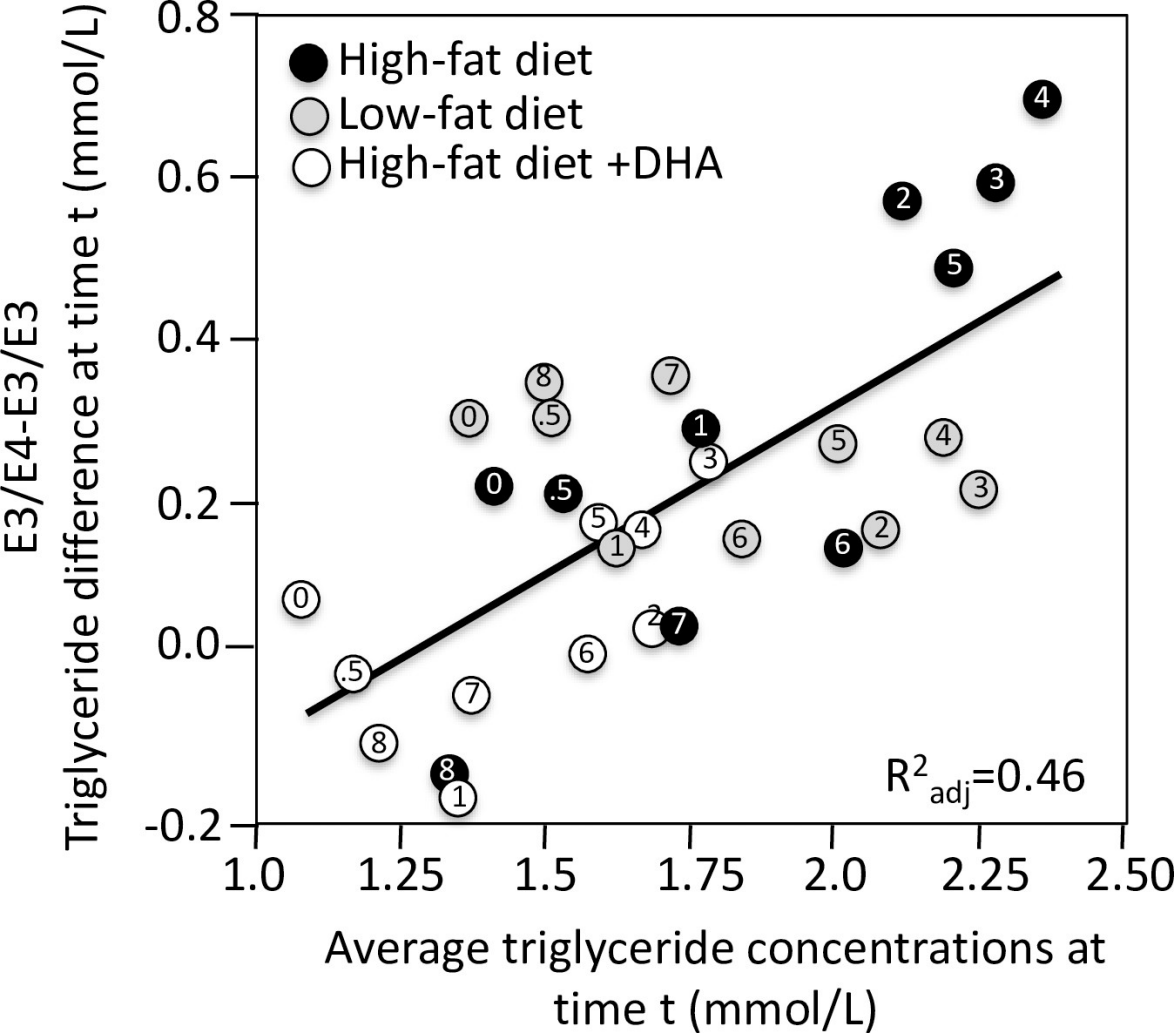
Quantile-dependent expressivity plots for postprandial triglyceride responses by *APOE* polymorphisms and diet. Derived from the postprandial response figures published by Jackson et al. for differences between 11 APOE E34 vs. 12 E33 men on low-fat diet; high saturated-fat diet; and high saturated-fat diet with fish oil [53].

In addition to these four important examples, significant quantile-dependent expressivity is suggested across a broad spectrum of other genetic variants affecting postprandial lipemia. Those achieving P≤0.05 significance are presented in Figs 4–7. Included among these are variants affecting secretion, lipolysis, and clearance, including *APOB* insertion/deletion (rs172404441, Fig 4A and 4B), familial hypobetalipoproteinemia cases with truncated apoB (Fig 4C), *APOB* L343V mutation (Fig 4D), *APOB* R463W substitution (Fig 4E), *SORT1* (Fig 4F), *APOA4* Q360H substitution (Fig 4G) and 347 Ser mutation (Fig 4H), Apo A-1_Milano_ (Fig 5A), *APOA1*–2803 polymorphism (Fig 5B), cholesterol ester transfer protein (*CETP*) isoleucine 405 to valine substitution (I405 → V) in exon 14 (Fig 5C), CETP deficiency (Fig 5D), Tangier disease (Fig 5E), *ABCA1* i48168 (rs4149272, Fig 5F) and i27943 genetic variants (rs2575875, Fig 5G), the rs7903146C/T polymorphism in Transcription factor 7–like 2 (*TCF7L2*, Fig 5H), rs1260326/P446L polymorphism of the glucokinase regulatory protein gene (*GCKR*, Fig 6A), the D314A mutation of the *GALNT2* gene which codes the UDP-N-Acetyl-D-galactosamine:polypeptide N-Acetylgalactosaminyl-transferase 2 enzyme (Fig 6B), the common leptin receptor (*LEPR*) Gln223Arg polymorphism (rs1137101, Fig 6C), the rs1800629 (-308G>A) polymorphism in the promoter region of tumor necrosis factor-alpha gene (*TNFA*, Fig 6D), the fatty acid transport protein 6 (*FATP6*)–7T>A polymorphism (rs2526246, Fig 6E), Mature Onset Diabetes of the Young type 3 (Fig 6F), the rs12970134 polymorphism near the melanocortin-4 receptor gene (*MC4R*, Fig 6G), the -1473G/C polymorphism of the interleukin 1 beta gene (*IL1b*, rs1143623, Fig 6H), the transmembrane 6 superfamily member 2 (*TM6SF2*) loss-of-function variant (rs58542926, Fig 7A), the -493G>T polymorphism in the promoter region of the microsomal triglyceride transfer protein (*MTTP*, rs1800591, Fig 7B), the Leu7Pro polymorphism of the neuropeptide Y (NPY) gene (Fig 7C); the lectin-like oxidized LDL receptor-1 (*LOX-1*) IVS4-14 A/G polymorphism (Fig 7D), and the angiopoietin-like protein 4 (ANGPTL4) T266M SNP (rs1044250, Fig 7E). Other example of quantile dependence are examined in the Discussion Section in relation to sex, age, disease, treatment and diet: *APOE* (Figs 7F, 9 and 13), *APOA5* (Figs 8 and 10), *FABP2* codon 54 (Fig 11), *SULF2* rs2281279 polymorphism (Fig 11), and *TCF7L2*, *TM6SF2*, and *MTTP* (Fig 12).

Twenty-six other reports did not provide significant evidence for quantile-dependent expressivity. Twelve of these were not actually negative results because they reported no significant effect of genotypes on postprandial triglyceride in their original publications and therefore could not be expected to provide evidence for quantile dependence, i.e., Byrne et al. for the *APOB* insertion/deletion polymorphism [12], Fisher et al. for *APOA4* Gln360His [33], Gerdes et al. analysis of *LPL* D9S [38], Jansen et al. for the C-480T transition in the *HL* promoter [57], Jayewardene analysis of CD36 gene polymorphisms [58], Masana et al. for *APOA 5*-1131T>C [72], Mooij et al. for hereditary multiple exostosis [77], Nierman et al. for *LPL* S447X [84]; Ostos et al. analyses of *APOA4* Gln360His [92], Pratley et al. for FABP2 Ala54Thr [101], Tahvanainen et al. for FABP2 Ala54Thr [116], and Tilly-Kiesi et al. for apoA-1 deletion of the codon for Lys 107 [121]. The remaining 14 papers showed limited or no statistically significant evidence for quantile dependence because of their limited statistical power, or lack of effect: Delgado-Lista et al. for *APOC3* at binding site -640 (P = 0.85) [27], Gerdes et al. for *LPL* N291S (P = 0.48) [38], Gertow et al. for *FATP1* intron 8 G/A polymorphism (P = 0.10) [39], Gudnason et al. for *CETP* Taq1B polymorphism (P = 0.11) [42], Jang et al. for *APOA5* -1131T>C (P = 0.17) [56], Kolovou et al. for *CETP* Taq1B polymorphism (P = 0.47) [62], Gomez-Delgado et al. for TNF-alpha rs1800629 (P = 0.08) [41], Martin et al. for *APOA5* S19W (P = 0.17) and *APOA5* -1131T>C (P = 0.11) [71], Masuda et al. for *CD36* deficiency (P = 0.72) [73], Mero et al. for *LPL* Asn291Ser (P = 0.76) [75], Miesenböck et al. for *LPL* missense mutation at codon 188 (P = 0.41) [76], Ooi et al. for *PCSK9* loss of function carriers (P = 0.35) [89], and Perez-Martinez et al. for the *APOB* -516C/T polymorphism (P = 0.07) [94]. Contrary to quantile dependent expressivity, Carpentier et al. report that 3 lipoprotein lipase deficient individual with extreme phenotype (fasting triglycerides >18 mmol/L) showed significantly smaller effect size when the controls postprandial triglycerides were highest [17].

## Discussion

Genetic variants are traditionally characterized by a fixed effect size, whereas the analyses presented in this report show that the effect size for the majority of genes affecting plasma triglyceride concentrations increase as plasma concentrations increase in the postprandial state. This phenomenon, quantile-dependent expressivity, may arise because the impaired functionalities of these genetic variants increase at higher triglyceride concentrations. These genetic variants showed a progressively increasing dose-response, with intermediate effect sizes at intermediate triglyceride concentrations. Postprandial observations are particularly compelling arguments for quantile-dependent expressivity because they demonstrate the phenomenon when triglyceride levels are manipulated within individuals, while factors contributing to the substantial between-person variability in lipemic response remain constant.

Quantile-dependent expressivity affects biological interpretation. Factors affecting plasma triglyceride concentration (e.g., sex, drugs, disease, diet, age [135]) will appear to interact significantly with genetic variants, leading to conclusions of gene-environment interactions and genetic predictors of drug efficacy (i.e., personalized medicine). Examples to follow show that such results may be more simply explained by the factors’ effects on triglyceride concentrations, which in turn change the genotype’s effect size in accordance with quantile-dependent expressivity.

### Sex differences

Olano-Martin et al.’s report on *APOA5*–1131 T>C polymorphism [88], Jackson et al.’s report on the *LEPR* Gln223Arg polymorphism (rs1137101) [52], Vimaleswaran et al. ‘s report on the *APOB* insertion/deletion polymorphism (rs17240441) [124], and Swatwan et al. reported on *LPL* S447X polymorphism [110] all hypothesize sex-dependent genetic effects. However, males are reported to have 63% higher fasting triglycerides, 61% higher maximum concentrations during the postprandial period, 63% greater area under the curve (AUC), and 77% greater incremental AUC when fasting triglycerides are subtracted (IAUC) [136]. All four genetic variants show strong dependence on total triglyceride concentrations during lipemia. Thus, quantile-dependent expressivity would predict a greater difference between genotypes in males than females in the postprandial state, as observed.

As a specific example, Fig 8 (upper panel) shows the TC vs. TT postprandial triglyceride difference for the *APOA5*–1131 T>C polymorphism in men and women [88]. The sex differences were originally attributed to the effects of sex steroids on receptor- and nonreceptor-dependent stages in TRL metabolism. The quantile-dependent expressivity plot in the lower panel shows that males and females represent largely nonoverlapping range of values over which the mean triglyceride concentrations predict increasingly larger TC vs. TT postprandial triglyceride differences. The graph clearly ascribes the difference between the sexes to male-female differences in plasma triglyceride levels and a shared (i.e., non-sex specific) underlying relationship between the effect size and overall average triglyceride levels.

### Fenofibrate treatment

Fenofibrate is a highly effective triglyceride-lowering treatment [137]. The effects of fenofibrate on postprandial triglycerides have been reported by apo E isoforms [51], −1131T>C *APOA5* polymorphism [16], *APOA5* 56G carriers vs. noncarriers [63], S19W polymorphism in *APOA5* [114], and the exon 1 G2S variant of the *SCARB1* gene [66]. All five studies concluded that the genotype predicted the efficacy of fenofibrate treatment to lower postprandial triglyceride concentrations.

Using data presented by Irvin et al. [51], the histogram in Fig 9 (upper panel) was created showing that fenofibrate-induced reductions in mean plasma triglyceride concentrations were greater in E4-carriers and E2-carriers than E33 homozygotes. This was true at fasting (time 0) and postprandially at 3.5 and 6 hours. This histogram of the treatment effect by genotype ignores the mean triglyceride concentrations pre- and post treatment, which are displayed in the middle panel. This middle panel emphasizes the difference between genotypes with arrows connecting the mean triglyceride concentrations from the E33 to the E2 carriers (E4-carriers always had an intermediate concentration). The far right section within the middle panel combines the arrows without regard to postprandial time. It shows that the genotype differences increase with average triglyceride concentrations, as illustrated by the quantile dependent expressivity plot of the E2-E33 effect size (dependent variable) vs. average triglyceride concentrations (independent variable) in the bottom panel. These analyses show an underlying relationship between the genetic effect size and average triglyceride concentration (bottom panel) that produces the difference between E2 and E33 genotypes (upper panel) when average triglyceride concentrations change in response to fenofibrate and fat ingestion (middle panel).

Fig 10 repeats these analyses for the results presented by Cardona et al. [16] and Lai et al. [63], showing: 1) genotype-specific mean reductions in fasting and postprandial triglycerides from fenofibrate treatment (left column), 2) different genetic effect sizes by fenofibrate use and postprandial status (center column), and 3) the underlying relationship between the genetic effect size (dependent variable) and average triglyceride concentration (independent variable) in the quantile-dependent expressivity plots (right column). Thus, each case suggests an underlying relationship between the genetic effect size and average triglyceride concentration (right column) that produces the difference between genotypes (left column) when average triglyceride concentrations change in response to fenofibrate and fat ingestion (center column).

Quantile-dependent expressivity provides a very different conceptual framework affecting the translation of these findings to clinical practice. There are two different interpretations to Figs 7 and 8: 1) the genetic variant predicts the change in postprandial lipemia (personalized medicine perspective represented by the histograms), and 2) postprandial triglyceride concentrations predict the effect size of the genetic variant (quantile-dependent expressivity). Whereas, some advocate individualized drug prescriptions through the use of genetic markers to identify patients most likely to benefit from fenofibrate treatment [138], quantile-dependent expressivity postulates that the results represent a basic phenomenon where the genetic effect size increases with plasma triglyceride concentration.

### Disease conditions

Metabolic syndrome, type 2 diabetes mellitus (T2DM), non-alcoholic fatty liver disease (NAFLD) and nonalcoholic steatohepatitis (NASH, NAFLD patients with inflammation) are all conditions known to increase plasma triglyceride concentration in fasting and postprandial states [135].

Fig 11 (upper panel) present apparent differences between T2DM and non-T2DM patients that we attribute to quantile-dependent expressivity. The first example involves the Fatty Acid–Binding Protein 2 (*FABP2*) gene codon 54, which produces a Thr-containing (mutated-type) intestinal fatty acid binding protein that has 2-fold greater affinity for long-chain fatty acids than the wild type Ala-containing protein. This mutation is hypothesized to increase intestinal absorption and processing of fatty acids leading to increased postprandial triglycerides observed in three studies [4,37,44]. Fig 11A shows the effect size increased with increasing average triglyceride concentrations separately within non-diabetics and diabetic patients, and for the patient populations combined. Thus, the apparent difference between T2DM and nonT2DM is consistent with their different triglyceride concentrations in the context of the gene’s greater effect size at higher triglyceride concentrations.

The second example involves the sulfate glucosamine-6-O- endosulfatase 2 (*SULF2*) gene, which is thought to play a role in hepatic clearance of postprandial remnants [43]. Fig 11B shows that carriers of the minor G allele of the *SULF2* rs2281279(A>G) SNP had lower postprandial triglycerides. Again, the different effect size in T2DM [43] than nonT2DM patients [74] was consistent with quantile-dependent expressivity and the difference in average triglyceride levels between T2DM vs. nonT2DM. Fig 12 presents three examples where apparent differences between patients with nonalcoholic fatty liver disease (NAFLD) and healthy controls can be attributed to quantile-dependent expressivity. Musso et al. reported that the transmembrane 6 superfamily member 2 (*TM6SF2*) rs58542926 polymorphism [83] and the transcription Factor 7–Like 2 (*TCF7L2*) rs7903146 polymorphism [81] had no effect on fasting plasma triglyceride concentrations. However, both polymorphisms affected postprandial triglyceride concentrations in patients with NAFLD. In NASH patients, carriers of the T allele of TCF7L2 showed significantly greater increases in postprandial plasma triglycerides than CC homozygotes, and the difference between genotypes in NASH patients was significantly greater than the difference in healthy patients (Fig 12A). In NAFLD patients, CC homozygotes of TM6SF2 showed significantly greater increases in postprandial plasma triglycerides than carriers of the T allele, and the difference between genotypes in NAFLD patients was again significantly greater than the difference in healthy patients (Fig 12B). The combined patient data show that for both polymorphisms, the difference between genotypes increased linearly with increasing average triglyceride concentrations, with the healthy patients clustered in the lower left quadrant and the NASH and NAFLD patients distributed along the diagonal (lower panels). The quantile-dependent expressivity interpretation is that the genetic effect size of the each polymorphism increases with increasing triglyceride concentrations, with NAFLD patients occupying different portions of the underlying triglyceride distribution (higher triglyceride) than healthy patients (lower triglycerides).

The third example involves the microsomal triglyceride transport protein’s lipid transfer activity that is required to lipidate and assemble chylomicrons, VLDL and LDL. Inhibition of the protein leads to decreased hepatic VLDL triglyceride secretion and triglyceride accumulation in hepatic cells leading to hepatic steatosis. The functional -493G->T polymorphism (rs1800591) occurs in the *MTTP* gene’s promoter region. Gambino et al. reported that carriers of the T allele had lower incremental area under the curve (iAUC) for triglycerides despite slightly higher fasting triglycerides in both healthy and NASH patients [35]. Fig 12C shows a somewhat greater increase in the triglyceride difference between genotypes with increasing triglyceride concentrations, with similar effects in healthy and NASH patients except for a larger genotype difference for NASH patients in accordance with their high average postprandial triglycerides.

### Diet

Consistent with the quantile-dependence expressivity of Fig 2, Fig 13 shows that the triglyceride differences between *APOE* E3/E4 and E3/E3 genotypes tend to be intermediate on a low fat diet (when plasma and postprandial triglycerides were intermediate), highest on a high fat, high-saturated fat diet (corresponding to higher average triglyceride concentrations), and lowest on a high fat, high-saturated fat diet consumed with 3.45 g/day docosahexaenoic acid (corresponding to lowest average triglyceride concentrations).

### Limitations

In almost all cases, the data were extracted using the vertical dimension of lines superimposed on figures that were imported into a computer-drawing program (Microsoft Powerpoint). This, no doubt, introduced error from both the original author’s rendering of the figures and my drawing of lines to extract their numerical data. The t-test for the linear regression slope should include these sources of measurement error. Regression analysis was performed separately from data extraction to ensure their independence. Approximately eighteen percent of the figures did not include standard errors, and those that did seemed less exactly drawn than the genotype-specific means themselves. Therefore, no effort was made to use the supplied standard errors to further improve upon the test for significant regression slopes. It is the author’s belief that the simple regression analyses presented in the figures is likely robust given that the fitted points are the average of multiple observations, and the genetic makeup of the sample did not change during the oral fat tolerance test. The extracted data are included as supplementary information that will hopefully motivate alternative analyses by others. My use of published results will certainly include publication bias in that there is little motivation for publishing nonsignificant results and that the vast majority of genotype data goes unreported for nonsignificant results. However, it is unlikely that publication bias affected the test of quantile-dependence given the hypothesis was heretofore largely unknown.

The analyses presented in this manuscript are not proposed as an alternative to the repeated measures analysis of variance or linear mixed models used in the studies identified by Parnell and colleagues [139,140]. Those analyses are designed test whether the genotypes affect the mean levels and the time course of the postprandial lipemia responses by genotype. The examples presented herein were selected on the basis of the repeated measures analyses attaining the statistical significance required to warrant publication, and nothing in our analyses raises questions about the validity of those original findings. Several of the included examples tested whether environmental factors significantly affect the genotypic postprandial lipemia response, as evidence for gene-environment interactions. Again, the analyses of this report do not challenge the statistical significance of the environmental effect.

The current analyses represent a post-hoc test of a very different question, whether the difference between genotypes increases linearly in association with mean plasma triglyceride concentrations. as a test of quantile-dependent expressivity. Biological explanations of gene-environment interactions traditionally assume epigenetic processes [139]. Quantile-dependent expressivity proposes that for genetic effects that are quantile dependent, environmental factors that distinguish high from low triglyceride concentrations will create a statistically significant environment-genotype interaction.

### Conclusion

Quantile-dependent expressivity applies to the majority of genetic variants affecting postprandial triglycerides. It provides an alternative explanation for sex, disease, and dietary interactions with genotype, and an alternative explanation to genetic markers for fenofibrate efficacy. Other results are fortified by controlling for quantile-dependent expressivity, such as Carvalho-Wells et al. claim that APOE genetic variants had a greater effect on postprandial triglycerides in older than younger patients (Fig 7F) [18]. Elsewhere it has been shown that quantile-dependent expressivity affects the genetic determination of other phenotypes (body mass index, HDL-cholesterol, LDL-cholesterol, fasting glucose concentrations) [3], that heritability of coffee consumption is quantile specific [141], and that quantile effects may partially explain the obesity epidemic affecting Western societies [142].

## Methods

The analyses presented in this paper are based exclusively on the published graphs of postprandial triglyceride responses over time. Of the 128 published papers, we identified 97 papers providing plots of postprandial triglyceride lipoprotein differences between genotypes at four or more time points (S1 Table). The figures were imported from the articles’ pdf files into Microsoft Powerpoint to extract their quantitative information (version 12.3.6 for Macintosh computers, Microsoft corporation, Redmond WA). For each figure, vertical lines were drawn to correspond to the overall height of the Y-axis, and the vertical distances between the X-axis and each plotted point. Their heights were recorded from the software’s formatting pallet and the individual plotted points were converted into concentrations based on the relative heights of the Y-axis and the plotted points (88.5 mg/dl = 1 mmol/L). The resultant dataset is provided as supplementary material (S1 Data). For each published figure, plots were created for the genetic effect by average triglyceride concentrations at each time point. Except where noted (Figs 2 and 11), the regression slopes were calculated within each study. In most cases the genetic effect was calculated as the difference between two genotypes with the heterozygote combined with one of the homozygotes, in other cases it was estimated from least squares regression as the average effect per dose of the higher-valued allele. Within each figure, the average triglyceride concentration at each time point “t” was calculated from triglyceride averages and sample sizes of the genotype-specific means. Specifically, if genotype “1” had a frequency of P_1_ and an average triglyceride of X_1(t)_ and genotype “2” a frequency of (1- P_1_) and an average triglyceride of X_2(t)_ then the average triglyceride for the total sample at time t was P_1_*X_1(t)_ + (1-P_1_)*X_2(t)_. For three genotypes with frequencies of P_1_, P_2_, and 1-P_1_-P_2_ and average triglycerides of X_1(t),_ X_2(t)_ and X_3(t)_, respectively, the average triglycerides at time t was calculated as P_1_*X_1(t)_ + P_2_*X_2(t)_ + (1-P_1_-P_2_)*X_3(t)_. In the case of a rare genotype vs. unaffected controls, the average triglyceride concentration was taken as the mean triglyceride concentration of the unaffected controls.

Linear regression analyses of the genetic effect (dependent variable) versus mean triglyceride concentrations (independent variable) were performed using JMP (version 5.1, SAS institute, Cary North Carolina). The regression models are based on the mean triglyceride values presented in the published figures and not individual subject responses. Adjusted coefficients of determination (R^2^_adj_) are presented to assess the level of correspondence between the average triglyceride differences between genotypes vs. average triglyceride concentrations at each time point, including the fasting (baseline) value. Although all of the regression models include only one explanatory variable, the adjusted R^2^ was used to penalize the R^2^ for the small number of observations (time points) used in the model. The slopes are presented with their standard error, and their significances based on the degrees of freedom (number of time points with fasting or postprandial measurements minus two).

The data is based on published summary reports that are publically available. A spreadsheet of the extracted quantified information by time and genotype are provided in supplementary material (S1 Data).

## Supporting information

S1 Table Studies identified as providing graphs of the postprandial triglyceride response. (DOCX)

S1 Data Data extracted from published postprandial lipemia graphs. (XLSX)
