# Supplementary material for: Quantile-dependent expressivity of postprandial lipemia
Source: PLoS One. 2020 Feb 26;15(2):e0229495. doi: 10.1371/journal.pone.0229495 (PMC7043740; doi:10.1371/journal.pone.0229495)
Supplement: S1 Table — (DOCX) [file pone.0229495.s001.docx]

| Table 1. Studies identified as providing graphs of the postprandial triglyceride response. | | | | | |
| --- | --- | --- | --- | --- | --- |
| Paper | Excl | Genetic variant | Dependent variable | Regression slope±SE | R^2^ |
| Agren 1998 [4] |  | FABP2 | ThrThr-AlaAla | 0.683±0.138  P=0.003 | 0.77 |
| Anagnostopoulou 2009 [5] | E1 |  |  |  |  |
| Auinger 2012 [6] |  | FATP6 (rs2526246) | T-carriers - AA | 0.103±0.028  (P=0.005) | 0.55 |
| Bergeron 1996 [7] |  | APOE | E34-E33 | 0.023±0.083  (P=0.80) | <0 |
| Berthier 2001 [8] | E3 |  |  |  |  |
| Boerwinkle 1994 [9] | E4 | APOE |  |  |  |
| Brenninkmeijer  1987 [10] |  | APOE | nonE2-E2 | 0.889±0.217  (P=0.009) | 0.72 |
| Brown 1991 [11] |  | APOE | E32-E33 | 0.055±0.168  (P=0.75) | <0 |
|  |  |  | E4_-E33 | 0.173±0.131  (P=0.23) | 0.08 |
| Byrne 1996 [12] |  | apo B signal peptide | ID/DD-II | 0.016±0.092  (P=0.87) | <0 |
| Calabresi 1993 [13] |  | Apo A1Milano | Milano - normals | 1.772±0.398  (P=0.007) | 0.76 |
| Cardona 2005 [14] | E3 |  |  |  |  |
| Cardona 2006 [15] | E3 |  |  |  |  |
| Cardona 2009 [16] | E4 | *APOA5* x drug |  |  |  |
| Carpentier 2012 [17] |  | LPL deficiency | Deficient-controls | -3.003±0.987  (P=0.02) | 0.54 |
| Carvalho-Wells 2010 [18] |  | APOE <50 | E2_-E33 | 0.368±0.068  (P=0.0004) | 0.74 |
|  |  |  | E4_-E33 | -0.039±0.024  (P=0.14) | 0.14 |
|  |  | APOE >50 | E2_-E33 | -0.047±0.051  (P=0.38) | <0 |
|  |  |  | E4_-E33 | 0.208±0.036  (P=0.0003) | 0.76 |
| Clemente-Postigo 2010 [19] | E3 |  |  |  |  |
| Connors 2014 [20] |  | SORT1 | TT-CT/CC | 0.349±0.070  (P=0.008) | 0.82 |
| Corella 2007 [21] | E2 |  |  |  |  |
| Dallongeville 1999 [22] |  | APOE | E2-E33 | -0.008±0.043  (P=0.86) | <0 |
|  |  |  | E4_-E33 | 0.067±0.049  (P=0.26) | 0.18 |
| Dart 1997 [23] |  | APOE | E4-noE4 | 0.331±0.108  (P=0.02) | 0.51 |
| Dart 1999 [24] | E4 |  |  |  |  |
| Delgado-Lista 2007 [25] |  | Apo A-II -265T/C | TT-CT/CC | 0.281±0.027  (P=1.5x10^-5^) | 0.93 |
| Delgado-Lista 2010 [26] |  | ABCA1 i48168 | CT/TT-CC | 0.480±0.079  (P=0.0005) | 0.82 |
|  |  | ABCA1 i27943 | A carriers vs. GG | 0.416±0.086  (P=0.002) | 0.74 |
| Delgado-Lista 2010 [27] |  | APOC3 -640 genotype | AA-CC/CA | 0.049±0.253  (P=0.85) | <0 |
|  |  | APOA1 - 2803 | GA - AA | 0.462±0.108  (P=0.004) | 0.68 |
|  |  | APOA4A5 inter SNP | TT – CC/CT | 0.364±0.051  (P=0.0002) | 0.86 |
|  |  |  |  |  |  |
| Delgado-Lista 2011 [28] |  | IL1B rs1143623 | GC/CC-GG | 0.432±0.068  (P=0.0004) | 0.83 |
| Engelbrechtsen 2017 [29] |  | TCF7L2 rs7903146 | CC-TT | 0.311±0.017  (P=3.4x10^-11^) | 0.96 |
| Erkkilä 2007 [30] |  | APOE week 0 | E22/E23-E33/ E34 | 0.537±0.128  (P=0.004) | 0.67 |
|  |  | APOE week 8 | E2_-E33 or E44 | 0.479±0.145  (P=0.01) | 0.55 |
| Fernández-Miranda 2001 [31] | E3 |  |  |  |  |
| Ferreira 2013 [32] |  | APOE sedentary | E2-E33 | 0.015±0.088  (P=0.87) | <0 |
|  |  | APOE moderate | E2-E33 | 0.015±0.096  (P=0.88) | <0 |
|  |  | APOE intensive | E2-E33 | -0.415±0.237  (P=0.18) | 0.34 |
|  |  | APOE sedentary | E4-E33 | 0.251±0.181  (P=0.26) | 0.19 |
|  |  | APOE moderate | E4-E33 | 0.653±0.380  (P=0.18) | 0.33 |
|  |  | APOE intensive | E4-E33 | 0.400±0.189  (P=0.12) | 0.46 |
| Fisher 1999 [33] |  | APOA4 His36Gln | GlnGln-  HisHis/HisGln | -0.080±0.099  (P=0.48) | <0 |
| Fontaine-Bisson 2007 [34] | E3 |  |  |  |  |
| Gambino 2007 [35] |  | MTP 493G/T  controls | GG-GT/TT | 0.075±0.372  (P=0.85) | <0 |
|  |  | MTP 493G/T  NASH | GG-GT/TT | 0.535±0.218  (P=0.07) | 0.50 |
|  |  | MTP 493G/T  combined | GG-GT/TT | 0.308±0.139  (P=0.05) | 0.26 |
| Geng 2018 [36] | E3 |  |  |  |  |
| Georgopoulos 2000 [37] |  | FABP2 | ThrThr-AlaAla | 0.927±0.401  (P=0.10) | 0.52 |
| Gerdes 1997 [38] |  | LPL D9N | N9-D9 | -0.017±0.032  (P=0.62) | <0 |
|  |  | LPL N291S | S291-N291 | 0.086±0.107  (P=0.48) | <0 |
| Gertow 2003 [39] |  | FATP1 | AA-AG/GG | 0.379±0.185  (P=0.10) | 0.35 |
| Gómez 2004 [40] |  | HL −514C/T | Per C-allele | 0.241±0.063  (P=0.007) | 0.63 |
| Gomez-Delgado 2014 [41] |  | TNF-alpha rs1800629 | GG-GA/AA | 0.038±0.014  (P=0.08) | 0.60 |
| Gudnason 1999 [42] |  | CETP Taq1B | B1B1-B1B2/B2B2 | 0.048±0.021  (P=0.11) | 0.50 |
|  |  | CETP I405V | II-IV/VV | 0.083±0.020  (P=0.03) | 0.80 |
| Hassing 2014 [43] |  | SULF2 rs2281279 | AA-AG/GG | 0.891±0.203  (P=0.007) | 0.75 |
| Helwig 2007 [44] |  | FABP2 Ala54Thr | ThrThr-Ala/Ala | 0.264±0.032  (P=2.2x10^-5^) | 0.86 |
| Hockey 2001 [45] |  | A-IV Q360H | A-IV-1/2 -  A-IV-1/1 | 0.538±0.088  (P=0.0005) | 0.82 |
| Holleboom 2011 [46] |  | GALNT2 D314A | Normal - mutation | 0.564±0.123  (P=0.006) | 0.77 |
| Hooper 2007 [47] |  | truncated apoB | Normal - truncated | 0.630±0.120  (P=0.006) | 0.84 |
| Hooper 2015 [48] |  | L343V FHBL | Normal vs. L343V | 0.708±0.167  (P=0.01) | 0.77 |
| Humphries 1998 [49] |  | LPL S447 and H X447 | S447-X447 | 0.159±0.058  (P=0.03) | 0.42 |
| Inazu 2008 [50] |  | CETP-deficient | Normal- deficient | 0.799±0.103  (P=0.02) | 0.95 |
| Irvin 2010 [51] | E4 | APOE x drugs |  |  |  |
| Jackson 2012 [52] |  | leptin receptor Gln223Arg | Per Gln dose | 0.169±0.023  (P=4.6x10-5) | 0.84 |
| Jackson 2012 [53] |  | APOE low fat | E4-E3 | -0.073±0.087  (P=0.43) | <0 |
|  |  | APOE high fat | E4-E3 | 0.607±0.139  (P=0.002) | 0.67 |
|  |  | APO E fish oil | E4-E3 | 0.332±0.154  (P=0.06) | 0.29 |
| Jackson 2016 [54] |  | TNFA -308 G>A | GG-GA/AA | 0.453±0.086  (P=0.001) | 0.77 |
| Jackson 2017 [55] | E2 |  |  |  |  |
| Jang 2004 [56] |  | APOA5 -1131T>C | CT/CC-TT | 0.167±0.092  (P=0.17) | 0.36 |
| Jansen 1999 [57] |  | HL promoter C 480T | CT/TT-CC | 0.060±0.032  (P=0.16) | 0.38 |
| Jayewardene 2016 [58] |  | rs1984112 | AA-GA/GG | 0.378±0.280  (P=0.27) | 0.17 |
|  |  | rs1527479 | TC/CC-TT | 0.015±0.716  (P=0.99) | <0 |
| Jiménez-Gómez 2008 [59] | E3 |  |  |  |  |
| Kobayashi 2001 [60] |  | APOE | E34-E33 | 0.471±0.085  (P=0.03) | 0.91 |
| Kolovou 2003 [61] |  | Tangier disease | Affected - Normal | 4.500±0.484  (P=0.01) | 0.97 |
| Kolovou 2007 [62] |  | TaqIB | B1B1-B1B2/B2B2 | 0.231±0.283  (P=0.47) | <0 |
| Lai 2007 [63] | E4 | APOA5 -1131T |  |  |  |
|  | E4 | APOA5 56G>C |  |  |  |
| Lai 2016 [64] | E3 |  |  |  |  |
| Lefevre 2005 [65] | E3 |  |  |  |  |
| Liu 2008 [66] | E2 |  |  |  |  |
| López-Miranda 1997 [67] |  | apo B XbaI | (X-X-) – (X+X+/X+X-) | 0.145±0.064  (P=0.058) | 0.34 |
| López-Miranda 2004 [68] |  | LPL HindIII S447X | H2S447 - H1X447 | 0.354±0.074  (P=0.002) | 0.73 |
| Lundahl 2002 [69] |  | MTP 493 | TT/TG-GG | 0.248±0.076  (P=0.02) | 0.61 |
| Marín 2002 [70] | E2 |  |  |  |  |
| Martin 2003 [71] |  | APOA5 S19W | SW-SS | 0.086±0.048  (P=0.17) | 0.36 |
|  |  | APOA5 -1131T>C | TC-TT | 0.091±0.041  (P=0.11) | 0.49 |
| Masana 2003 [72] |  | APOAV – 1131T>C | TT-CT/CC | 0.558±0.235  (P=0.08) | 0.48 |
| Masuda 2009 [73] |  | CD36 deficiency | Normal-deficient | 0.519±1.346  (P=0.72) | <0 |
| Matikainen 2013 [74] |  | SULF2 rs2281279 | AA-GG/GA | 0.133±0.192  (P=0.54) | <0 |
| Mero 1999 [75] |  | LPL Asn291Ser | Ans291Ser-control | 0.086±0.251  (P=0.76) | <0 |
| Miesenböck 1993 [76] |  | LPL codon 188 | Mutant-controls | 0.400±0.431  (P=0.41) | <0 |
| Mooij 2015 [77] |  | Her multiple exostosis | HME-controls | -0.006±0.154  (P=0.97) | <0 |
| Moreno 2003 [78] | E3 |  |  |  |  |
| Moreno 2006 [79] |  | APOAV – 1131T>C | CC/CT- TT | 0.193±0.084  (P=0.055) | 0.35 |
| Moreno-Luna 2007 [80] |  | APOA5 | Homozygotes-other | 0.305±0.102  (P=0.02) | 0.50 |
| Musso 2009 [81] |  | TCF7L2 C/T | CT/TT-CC | 0.507±0.123  (P=0.01) | 0.76 |
| Musso 2011 [82] |  | LOX-1 | AA-GA/AA | 0.261±0.063  (P=0.01) | 0.76 |
| Musso 2017 [83] |  | TM6SF2 C>T | CC - TT/TC | 0.641±0.082  (P=1.5x10-5) |  |
|  |  |  |  |  |  |
| Nierman 2005 [84] |  | LPLS447X | S447X-control | 0.207±0.09  (P=0.09) | 0.55 |
| Nikkilä 1994 [85] |  | Apo E cases | E32-E33 | 0.237±0.472  (P=0.67) | <0 |
|  |  | Apo E controls | E32-E33 | 0.437±0.155  (P=0.11) | 0.70 |
|  |  | Apo E cases | E34-E33 | 0.344±0.162  (P=0.17) | 0.54 |
|  |  | Apo E controls | E34-E33 | -0.175±0.117  (P=0.27) | 0.29 |
| Noto 2009 [86] |  | APOB R463W | Normal -R463W | 0.719±0.054  (P=9.1x10-7) | 0.95 |
| O’Hare 2017 [87] |  | TM6SF2 rs58542926 | CC-TC/TT | 0.071±0.025  (P=0.05) | 0.58 |
| Olano-Martin 2008 [88] |  | APOA5 -1131 males | CC/CT-TT | 0.279±0.053  (P=0.0005) | 0.73 |
|  |  | APOA5 -1131 females | CC/CT-TT | 0.135±0.072  (P=0.09) | 0.20 |
| Ooi 2017 [89] |  | PCSK9 LoF | Normal-LoF | 0.170±0.138  (P=0.35) | 0.14 |
| Orth 1996 [90] | E2 |  |  |  |  |
| Ostos 1998 [91] |  | apoA-IV-347Ser | ThrThr -  SerSer/SerThr | 0.299±0.108  (P=0.03) | 0.46 |
| Ostos 2000 [92] |  | A-IV- Gln360His | HisHis/HisGln  -Gln/Gln | 0.083±0.074  (P=0.30) | 0.03 |
| Pérez-Martínez 2004 [93] |  | SR-B1 | 1/2-1/1 | 0.046±0.119  (P=0.71) | <0 |
| Pérez-Martínez 2007 [94] |  | APOB-516C/T | CT-CC | 0.273±0.129  (P=0.07) | 0.30 |
| Pérez-Martínez 2008 [95] | E3 |  |  |  |  |
| Pérez-Martínez 2009 [96] | E4 | GCKR-APOA5 x drug |  |  |  |
| Pérez-Martínez 2011 [97] |  | melanocortin-4 receptor | GG-GA/AA | 0.211±0.074  (P=0.02) | 0.47 |
| Pérez-Martínez 2012 [98] | E3 |  |  |  |  |
| Pimstone 1996 [99] |  | LPL Asn291Ser | Mutant-control | 2.027±0.374  (P=0.003) | 0.83 |
| Pollin 2008 [100] |  | ApoC3 R19X | CC-CT | 0.498±0.018  (P=1.1x10-5) | 0.99 |
| Pratley 2000 [101] |  | FABP2 amino acid 54 | Thr54-Ala54 | 0.006±0.080  (P=0.94) | <0 |
| Regis-Bailly 1996 [102] | E2 |  |  |  |  |
| Reiber 2003 [103] |  | LPL HindIII | H+H+/H+H- –  H-/- | 0.580±0.102  (P=0.005) | 0.86 |
|  |  | APOE | E34-E33 | 0.743±0.186  (P=0.02) | 0.75 |
| Reznik 1996 [104] |  | APOE | E23-E33 | -0.238±0.076  (P=0.02) | 0.56 |
|  |  |  | E34-E33 | -0.435±0.077  (P=0.001) | 0.82 |
| Reznik 2002 [105] | E3 |  |  |  |  |
| Ribalta 2005 [106] | E3 |  |  |  |  |
| Rubin 2008 [107] | E3 |  |  |  |  |
| Saleheen 2017 [108] |  | APOC3 LoF | Normal-LoF | 0.782±0.119  (P=0.02) | 0.93 |
| Schwab 2002 [109] |  | Neuropeptide Y Leu7Pro | LeuPro-LeuLeu | 0.313±0.115  (P=0.03) | 0.48 |
| Shatwan 2016 [110] | E3 |  |  |  |  |
| Shen 2008 [111] | E4 | IL5 -174C/G  (rs1800795) |  |  |  |
| Shen 2009 [112] |  | GCKR P446L rs1260326 | TT-CT/CC | 0.200±0.024  (P=0.001) | 0.93 |
| Smart 2011 [113] | E3 |  |  |  |  |
| Smith 2008 [114] | E2 |  |  |  |  |
| St-Jean 2017 [115] |  | HNF1 MODY3 | Normal-MODY3 | 0.364±0.098  (P=0.004) | 0.51 |
| Tahvanainen 2000 [116] |  | FABP2 | ThrThr-ThrAla/AlaAla | 0.060±0.049  (P=0.30) | 0.12 |
| Talmud 1998 [117] |  | LPL pro-moter -93T/G | TD-GDGN | 0.253±0.032  (P=0.004) | 0.94 |
| Talmud 2008 [118] |  | ANGPTL4 T266M  (rs1044250) | Doses T allele | 0.072±0.016  (P=0.02) | 0.83 |
| Tanaka 2007 [119] | E3 |  |  |  |  |
| Tanaka 2007 [120] | E3 |  |  |  |  |
| Tilly-Kiesi 1998 [121] |  | apoA-I (Lys107 0) | Unaffected-affected | 0.147±0.252  (P=0.59) | <0 |
| Vansant 1999 [122] |  | Apo E | E23-E33 | 0.176±0.137  (P=0.26) | 0.10 |
|  |  |  | E34-E33 | 0.287±0.061  (P=0.006) | 0.78 |
| van 't Hooft 2001 [123] | E2 |  |  |  |  |
| Vimaleswaran 2015 [124] |  | APOB ins/del rs17240441 | Per insertion dose | 0.350±0.046  (P=0.0001) | 0.88 |
| Warodomwichit 2009 [125] | E4 | TCF7L2 |  |  |  |
| Waterworth 1999 [126] |  | APOC3 T-2854G | GG-TT/TG | 0.192±0.045  (P=0.02) | 0.81 |
| Weintraub 1987 [127] | E2 |  |  |  |  |
| Wojczynski 2015 [128] | E4 |  |  |  |  |
| Wolever 1997 [129] |  | Apo E | E4 carrier-no E4 | 0.316±0.072  (P=0.002) | 0.64 |
| Woo 2003 [130] |  | CIII T2854G | GG-TT/TG | 0.470±0.139  (P=0.04) | 0.72 |
| Zemánková 2017 [131] |  | Apo A5 rs662799 & rs3135506 | Heterozygotes-wild | 0.868±0.124  (P=0.0004) | 0.87 |
| Exclusion codes: Sample bias due to selection for postprandial response (E1), total triglyceride results not provided in original paper (E2), total triglycerides presented as AUC, iAUC or pathological postprandial response (E3), total triglycerides presented for 3 time points (E4, included in data set and in the analysis of drug effect and pooled analysis of APOE with other samples but not other genetic variants). | | | | | |
